# Organ Dysfunction in Children With Blood Culture-Proven Sepsis: Comparative Performance of Four Scores in a National Cohort Study

**DOI:** 10.1097/PCC.0000000000003388

**Published:** 2023-10-25

**Authors:** Luregn J. Schlapbach, Sabrina Goertz, Niels Hagenbuch, Blandine Aubert, Sebastien Papis, Eric Giannoni, Klara M. Posfay-Barbe, Martin Stocker, Ulrich Heininger, Sara Bernhard-Stirnemann, Anita Niederer-Loher, Christian R. Kahlert, Giancarlo Natalucci, Christa Relly, Thomas Riedel, Christoph Aebi, Christoph Berger, Philipp K. A. Agyeman

**Affiliations:** 1 Department of Intensive Care and Neonatology, and Children`s Research Center, University Children`s Hospital Zurich, Zurich, Switzerland.; 2 Child Health Research Centre, University of Queensland, Brisbane, QLD, Australia.; 3 Division of Infectious Diseases, and Children’s Research Center, University Children’s Hospital Zurich, Zurich, Switzerland.; 4 Department of Pediatrics, Inselspital, Bern University Hospital, University of Bern, Bern, Switzerland.; 5 Clinic of Neonatology, Department Mother-Woman-Child, Lausanne University Hospital and University of Lausanne, Lausanne, Switzerland.; 6 Pediatric Infectious Diseases Unit, Department of Woman, Child and Adolescent, Children’s Hospital of Geneva, University Hospitals of Geneva and Faculty of Medicine, Geneva, Switzerland.; 7 Children’s Hospital Lucerne, Lucerne, Switzerland.; 8 Infectious Diseases and Vaccinology, University Children’s Hospital Basel, Basel, Switzerland.; 9 Children’s Hospital Aarau, Aarau, Switzerland.; 10 Children’s Hospital of Eastern Switzerland, St. Gallen, Switzerland.; 11 Department of Neonatology, University Hospital Zurich, Zurich, Switzerland.; 12 Department of Pediatrics, Cantonal Hospital Graubuenden, Chur, Switzerland.

**Keywords:** bacteremia, bacterial infections, child mortality, multiple organ failure, systemic inflammatory response syndrome

## Abstract

**OBJECTIVES:**

Previous studies applying Sepsis-3 criteria to children were based on retrospective analyses of PICU cohorts. We aimed to compare organ dysfunction criteria in children with blood culture-proven sepsis, including emergency department, PICU, and ward patients, and to assess relevance of organ dysfunctions for mortality prediction.

**DESIGN:**

We have carried out a nonprespecified, secondary analysis of a prospective dataset collected from September 2011 to December 2015.

**SETTING:**

Emergency departments, wards, and PICUs in 10 tertiary children’s hospitals in Switzerland.

**PATIENTS:**

Children younger than 17 years old with blood culture-proven sepsis. We excluded preterm infants and term infants younger than 7 days old.

**INTERVENTIONS:**

None.

**MEASUREMENTS AND MAIN RESULTS:**

We compared the 2005 International Pediatric Sepsis Consensus Conference (IPSCC), Pediatric Logistic Organ Dysfunction-2 (PELOD-2), pediatric Sequential Organ Failure Assessment (pSOFA), and Pediatric Organ Dysfunction Information Update Mandate (PODIUM) scores, measured at blood culture sampling, to predict 30-day mortality. We analyzed 877 sepsis episodes in 807 children, with a 30-day mortality of 4.3%. Percentage with organ dysfunction ranged from 32.7% (IPSCC) to 55.3% (pSOFA). In adjusted analyses, the accuracy for identification of 30-day mortality was area under the curve (AUC) 0.87 (95% CI, 0.82–0.92) for IPSCC, 0.83 (0.76–0.89) for PELOD-2, 0.85 (0.78–0.92) for pSOFA, and 0.85 (0.78–0.91) for PODIUM. When restricting scores to neurologic, respiratory, and cardiovascular dysfunction, the adjusted AUC was 0.89 (0.84–0.94) for IPSCC, 0.85 (0.79–0.91) for PELOD-2, 0.87 (0.81–0.93) for pSOFA, and 0.88 (0.83–0.93) for PODIUM.

**CONCLUSIONS:**

IPSCC, PELOD-2, pSOFA, and PODIUM performed similarly to predict 30-day mortality. Simplified scores restricted to neurologic, respiratory, and cardiovascular dysfunction yielded comparable performance.

**RESEARCH IN CONTEXT**

- Although sepsis is defined as infection with organ dysfunction, it is currently unclear which of the four available organ dysfunction criteria in children—2005 International Pediatric Sepsis Consensus Conference (IPSCC) criteria, Pediatric Logistic Organ Dysfunction-2 (PELOD-2), pediatric Sequential Organ Failure Assessment (pSOFA), and Pediatric Organ Dysfunction Information Update Mandate (PODIUM)—perform best for children with sepsis.
- While adult sepsis criteria are based on the SOFA score, pediatric sepsis remains defined by IPSCC criteria, which were crafted in 2005.
- In view of the planned revision of pediatric sepsis criteria, there is a need for robust evaluation of organ dysfunction score performance in children with confirmed sepsis.

**AT THE BEDSIDE**

- In this population-based study, IPSCC, PELOD-2, pSOFA, and PODIUM, assessed at blood culture sampling, achieved a similar prediction of 30-day mortality in children with proven sepsis.
- Although we observed substantial differences in individual adjudication of the type of organ dysfunction between scores, neurologic, respiratory, and cardiovascular dysfunction were consistently most relevant to predict 30-day mortality.
- Clinical assessment of neurologic, respiratory, and cardiovascular dysfunction at the bedside has the potential to enhance timely recognition of children with sepsis at risk of poor outcome.

Following the 1992 and 2001 consensus statement of the American College of Chest Physicians, pediatric sepsis was defined through expert opinion in 2005 by the International Pediatric Sepsis Consensus Conference (IPSCC) as infection in presence of at least two out of four criteria of systemic inflammatory response syndrome (SIRS) (1, 2). Severe sepsis was defined as sepsis with organ dysfunction, with more weight given to cardiovascular and respiratory dysfunction (3). These pediatric “Sepsis-2” definitions remain the only ones available for this age group, yet they have not been adequately validated (3).

The recent Sepsis-3 consensus definition demonstrated that infected adult patients with organ dysfunction, as measured by the Sequential Organ Failure Assessment (SOFA) score, had higher mortality than those without organ dysfunction, implying higher utility of the SOFA score compared with SIRS for mortality prediction (4). The 2020 pediatric Surviving Sepsis Campaign accordingly used the terms “sepsis-associated organ dysfunction” and “septic shock” to delineate sepsis, rather than “severe sepsis” (5). Given the limitations of the IPSCC, criteria for pediatric sepsis are currently being revised, and a global survey among pediatricians identified that the concept of sepsis-associated organ dysfunction is widely accepted to distinguish sepsis from infection (6–8). Yet, controversy surrounds the optimal operationalization of organ dysfunction scoring in children with infection (9). Most previous cohorts on pediatric sepsis included either children with suspected infections, lacking comprehensive microbiological diagnostics, or children with microbiological results that may represent either colonization, co-infection, or primary viral infection (10–14). Furthermore, most studies were performed in PICUs, and the generalization of findings to less acute cohorts, such as children in emergency department (ED) or ward settings, remains poorly understood (15, 16).

Therefore, we have used a curated dataset from 2011 to 2015 population-based multicenter prospective cohort study, with clearly defined, gold standard phenotype of blood culture-proven sepsis (2), to test the predictive performance of existing scoring systems to identify children at higher mortality risk.

## MATERIALS AND METHODS

### Study Design

We have carried out a nonprespecified secondary analysis of data collected by the Swiss Pediatric Sepsis Study, 2011–2015 (17). This prospective, observational multicenter cohort study investigated blood culture-proven sepsis, as defined by IPSCC definitions (2), in children in Switzerland. All ten major children’s hospitals in the country participated in the study. These sites accounted for 78% of all pediatric hospital admissions and 98% of all PICU admissions with an *International Classification of Diseases*, 10th Revision code for pathogen-specific sepsis in children in Switzerland (17). The current analysis was approved by the ethics committees of all participating centers (Cantonal Ethics Committee Bern, approval number KEK-029/11, approval date February 20, 2018, study title “Swiss Pediatric Sepsis Study—Impact of innate immunity on susceptibility to sepsis in neonates and children”) and the study was conducted in accordance with the ethical principles described in the Declaration of Helsinki. We followed the Strengthening the Reporting of Observational Studies in Epidemiology reporting guideline (18).

### Cohort Characteristics

In 2011–2015, infants and children presenting with blood culture-proven bacterial or fungal sepsis, as defined by IPSCC definitions (2), were eligible if they were recruited to the Swiss Pediatric Sepsis Study with an age between older than 7 days and younger than 17 years at sepsis onset and if patient files to verify organ dysfunction were available. We excluded preterm neonates born younger than 37 weeks and neonates with early-onset sepsis at younger than 7 days of life.

### Outcome

The primary outcome used in the current analysis was 30-day (in-hospital) mortality. The composite secondary outcome was defined as 30-day (in-hospital) mortality or PICU length of stay of 3 days or longer after blood culture sampling (19). Data on demographics, perinatal and other risk factors, comorbidities, infection site, severity, and outcome were entered prospectively into an online database. For the current analysis, we post hoc categorized comorbidities according to the pediatric complex chronic conditions classification system version 2 (20).

### Organ Dysfunction Scores

In the original study (17), investigators prospectively categorized every patient as having sepsis, severe sepsis, or septic shock, according to IPSCC criteria (2). For this curated dataset, we additionally collected the variables required to define, post hoc, individual organ dysfunctions according to IPSCC (2), Pediatric Logistic Organ Dysfunction-2 (PELOD-2) (21), pediatric SOFA (pSOFA) (22), and Pediatric Organ Dysfunction Information Update Mandate (PODIUM) (23) (**Supplementary Methods**, http://links.lww.com/PCC/C434). For every parameter, the worst value documented within the same calendar day during which the blood culture was obtained was collected. If a laboratory parameter had not been performed (such as lactate or arterial blood gas), it was assumed to be normal, which is aligned with contemporary scoring methodology (21). For PELOD-2, pSOFA, and PODIUM, in addition to using the full scores, we constructed a binary (yes/no) variable for the presence of each individual organ dysfunction, defined as yes if the score summary of all variables contributing to a given organ were greater than 0. Accordingly, we constructed five (PELOD-2), six (IPSCC and pSOFA), and eight (PODIUM) individual organ dysfunctions as binary variables.

### Statistics

Descriptive statistics are presented as median and interquartile ranges (IQRs) for continuous variables, and frequencies and percentages for categorical variables. We assessed the agreement between the four scores in relation to adjudication of specific organs affected by calculating the relative agreement and Krippendorff’s alpha, with 95% CIs derived from 5000 bootstrap samples (24). We considered a Krippendorff’s alpha greater than 0.80 as good and greater than 0.67 as acceptable agreement (24). We then assessed the discriminative power of the scores for the primary and secondary outcome using area under the curve (AUC) of receiver operating characteristic curves (25). To adjust the AUCs for age, sex, and presence of chronic medical conditions, we fitted logistic mixed-effects models with a random intercept for each hospital, using the linear predictor of the fixed effects for further analysis by receiver operating characteristic curves and corresponding AUCs. We estimated 95% CIs using DeLong’s method (26). We assessed the calibration of adjusted models with the Hosmer-Lemeshow C*-statistic (27) and Cox’s calibration regression (28), and the overall fit of the models with Brier score (29). We then examined the contribution of individual organs for the prediction of the primary and secondary outcome for the IPSCC, PELOD-2, pSOFA, and PODIUM scores, respectively. For this analysis, we used conditional random forest analyses that are unbiased in the presence of variables with different number of levels (30). The relevance of the individual organs within a score for the prediction of the outcome was judged by the permutation importance (31). We additionally analyzed the IPSCC and the binarized PELOD-2, pSOFA, and PODIUM scores with logic regression models that find the optimal combination of organs via cross-validation of logic trees. We did sensitivity analyses using only the first sepsis episode in every patient, to account for the fact that a patient could experience more than one sepsis episode. Further details on the statistical analyses are presented in the Supplementary Methods (http://links.lww.com/PCC/C434). All analyses were conducted with R Version 4.1.2 (32).

## RESULTS

### Cohort Description

We analyzed 877 sepsis episodes in 807 patients (**Fig. S1**, http://links.lww.com/PCC/C434) in which data were available. Seven hundred fifty-one patients experienced one sepsis episode, and 56 (6.9%) between two and six episodes. Overall, 357 of 877 episodes (40.7%) occurred in female children and the median age at sepsis was 32 months (IQR, 5–94 mo) (**eTable 1**, http://links.lww.com/PCC/C434). In 442 of 877 episodes (50.4%), patients were previously healthy, and 227 of 877 episodes (25.9%) were classified as hospital-acquired. The median length of stay in hospital was 14 days (IQR, 8–28 d). In 289 of 877 episodes (33.0%), admission to PICU was required with a median PICU length of stay of 7 days (IQR, 2–30 d). The primary outcome, 30-day (in-hospital) mortality, occurred in 38 of 877 episodes (4.3%), the secondary outcome, 30-day (in-hospital) mortality or PICU length of stay of 3 days or longer after blood culture sampling, in 226 of 877 episodes (25.8). The median time to death after obtaining the blood culture was 6 days (IQR, 1–16.5 d).

### Presence of Organ Dysfunction

**Figure 1.**
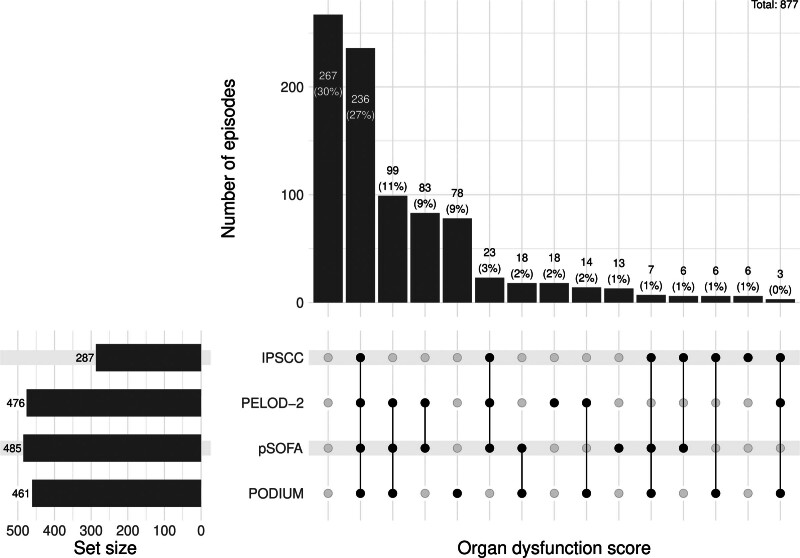
Upset demonstrating adjudication of presence of an organ dysfunction affected by each of the four organ dysfunction scores. Upset (43) displaying intersections of the four organ dysfunction scores (sets). The *lower* shows a matrix layout for all intersections, with *dark circles* indicating a sets is part of the intersection (i.e., presence of an organ dysfunction as defined by each of the four organ dysfunction scores). The *upper* shows the size of intersections. IPSCC = International Pediatric Sepsis Consensus Conference, PELOD-2 = Pediatric Logistic Organ Dysfunction-2, PODIUM = Pediatric Organ Dysfunction Information Update Mandate, pSOFA = pediatric Sequential Organ Failure Assessment.

Episodes of sepsis meeting criteria for organ dysfunction by score were: IPSCC in 287 of 877 episodes (32.7%); PELOD-2 in 476 of 877 episodes (54.3%); pSOFA in 485 of 877 episodes (55.3%); and PODIUM in 461 of 877 episodes (52.6%) (**eTable 2**, http://links.lww.com/PCC/C434). The mortality in children without organ dysfunction was less than 2.5% across all scores. The three most common organ systems affected were: cardiovascular, respiratory, and hematological for IPSCC; cardiovascular, renal, and hematological for PELOD-2; cardiovascular, neurologic, and hematological for pSOFA; and cardiovascular, hematological, and immunological for PODIUM. For cardiovascular dysfunction Krippendorff’s alpha was 0.47 (95% CI, 0.43–0.52), for respiratory dysfunction 0.79 (0.73–0.84), for neurologic dysfunction 0.68 (0.61–0.74), for renal dysfunction 0.52 (0.46–0.58), for hepatic dysfunction 0.25 (0.16–0.34), and for hematological dysfunction 0.73 (0.68–0.76) (eTable 2, http://links.lww.com/PCC/C434). The level of agreement between the four scores did not reach the threshold of good agreement for any organ dysfunction but was acceptable for respiratory and hematological dysfunction. The differences in adjudication of organs affected by the different scores are shown in **Figure 1**; and **Figure S2** (http://links.lww.com/PCC/C434).

**Figure 2.**
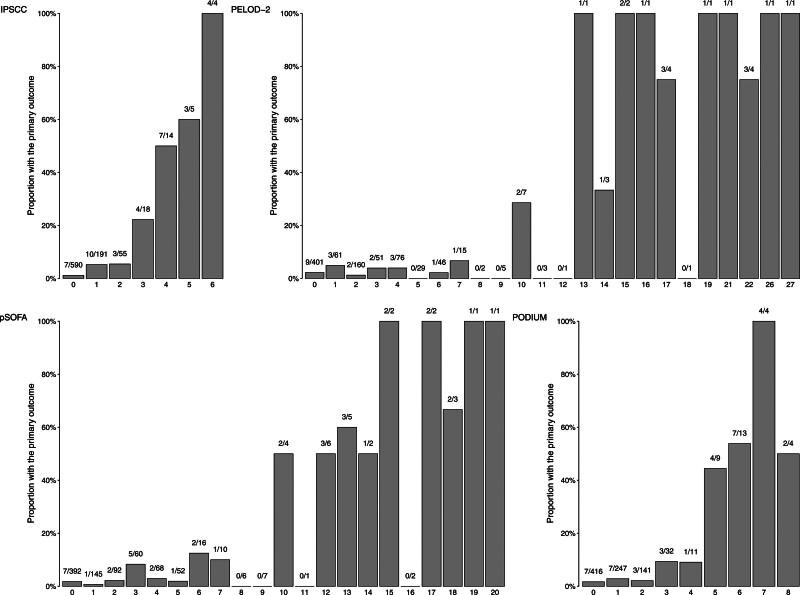
Proportions of patients meeting the primary outcome (30-d mortality) in relation to score value, according to the four organ dysfunction scores used. Proportion of episodes with the primary outcome for International Pediatric Sepsis Consensus Conference (IPSCC), Pediatric Logistic Organ Dysfunction-2 (PELOD-2), pediatric Sequential Organ Failure Assessment (pSOFA), and Pediatric Organ Dysfunction Information Update Mandate (PODIUM). The *x*-axes denote the number of organs affected (IPSCC) or the total score of each of the four organ dysfunction scores. *Numbers* above *bars* indicate number with the primary outcome/total number.

Across all four scores, mortality increased incrementally with higher score values (**Fig. 2**), as well as with higher number of organs affected (**Fig. S3**, http://links.lww.com/PCC/C434). Across all four scores, the proportion of children with the secondary outcome mortality and/or PICU length of stay of greater than or equal to 3 days increased incrementally with higher score values (**Fig. S4**, http://links.lww.com/PCC/C434), as well as with higher number of organs affected (**Fig. S5**, http://links.lww.com/PCC/C434).

### Receiver Operating Characteristics Curve Analysis With Primary and Secondary Outcomes

**Figure 3.**
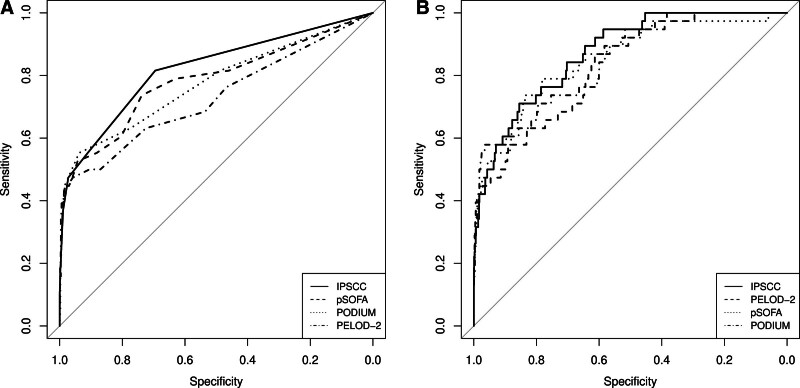
Area under the receiver operating characteristics curve analyses to predict the primary outcome (30-d mortality) for each of the four organ dysfunction scores used. Unadjusted (**A**), and analyses adjusted for age, sex, and presence of comorbidities, with a random effect per study site (**B**) are shown. IPSCC = International Pediatric Sepsis Consensus Conference, PELOD-2 = Pediatric Logistic Organ Dysfunction-2, PODIUM = Pediatric Organ Dysfunction Information Update Mandate, pSOFA = pediatric Sequential Organ Failure Assessment.

Discrimination of the primary outcome, 30-day mortality, by the organ dysfunction scores ranged between AUC 0.73 (95% CI, 0.63–0.83) for PELOD-2 and AUC 0.82 (95% CI, 0.74–0.90) for IPSCC (**Fig. 3*A***; and **eTable 3**, http://links.lww.com/PCC/C434). Discrimination was comparable for PELOD-2, pSOFA, and PODIUM in their binarized form (eTable 3 and **Fig. S6*A***, http://links.lww.com/PCC/C434).

Discrimination of the secondary outcome, 30-day (in-hospital) mortality or PICU length of stay of 3 days or longer after blood culture sampling, by the organ dysfunction scores ranged between AUC 0.67 (95% CI, 0.62–0.71) for PELOD-2 and AUC 0.71 (95% CI, 0.67–0.75) for pSOFA (eTable 3 and **Fig. S7*A***, http://links.lww.com/PCC/C434). Discrimination was comparable for PELOD-2, pSOFA, and PODIUM in their binarized form (eTable 3 and **Fig. S8*A***, http://links.lww.com/PCC/C434). Results were confirmed in sensitivity analyses (eTable 3, http://links.lww.com/PCC/C434).

### Adjusted Analyses With Primary and Secondary Outcomes

We then performed generalized linear mixed models adjusting for age, sex, and presence of comorbidities, with a random effect per study site. The baseline model (not including organ dysfunction scores) yielded an AUC of 0.67 (95% CI, 0.58–0.76) for the primary outcome, and an AUC of 0.59 (95% CI, 0.52–0.67) for the secondary outcome. In adjusted analyses, the discrimination of the primary outcome by the organ dysfunction scores ranged between AUC 0.83 (95% CI, 0.76–0.89) for PELOD-2 and AUC 0.87 (95% CI, 0.82–0.92) for IPSCC (**Fig. 3*B*** and **Table 1**). Model calibration and discrimination were comparable for PELOD-2, pSOFA, and PODIUM in their binarized form (Table 1; and **Fig. S6*B***, http://links.lww.com/PCC/C434).

**TABLE 1.** Measures of Calibration and Discrimination of Adjusted Models to Predict the Primary Outcome (30-d Mortality) in Children With Sepsis

Model Assessment	Original Scores				Binarized Scores		
	International Pediatric Sepsis Consensus Conference	PELOD-2	pSOFA	PODIUM	PELOD-2	pSOFA	PODIUM
Area under the curve (95% CI)	0.87 (0.82–0.92)	0.83 (0.76–0.89)	0.85 (0.78–0.92)	0.85 (0.78–0.91)	0.81 (0.74–0.88)	0.82 (0.74–0.89)	0.84 (0.77–0.90)
Hosmer-Lemeshow C*							
X^2^ (8)	10.28	10.98	4.37	20.69	24.18	6.80	19.31
*p*	0.25	0.20	0.82	0.008	0.002	0.56	0.013
Cox’s calibration regression							
Intercept (95% CI)	0.00 (–0.01 to 0.02)	0.01 (–0.01 to 0.02)	0.00 (–0.01 to 0.01)	0.00 (–0.02 to 0.02)	0.00 (–0.02 to 0.02)	0.00 (–0.01 to 0.02)	0.00 (–0.01 to 0.02)
Slope (95% CI)	0.99 (0.84–1.14)	0.86 (0.67–1.04)	0.97 (0.88–1.06)	0.98 (0.72–1.24)	0.99 (0.67–1.30)	0.96 (0.78–1.13)	0.94 (0.71–1.17)
X^2^ (1)	63.49	43.93	60.72	61.13	47.74	42.12	52.36
*p*	< 0.001	< 0.001	< 0.001	< 0.001	< 0.001	< 0.001	< 0.001
Brier score	0.03	0.03	0.03	0.03	0.03	0.03	0.03

PELOD-2 = Pediatric Logistic Organ Dysfunction-2, PODIUM = Pediatric Organ Dysfunction Information Update Mandate, pSOFA = pediatric Sequential Organ Failure Assessment.

Area under the curve of receiver operating characteristics curves are shown with respective 95% CIs. Analyses were adjusted for age (yr), sex, and presence of comorbidities, with a random effect per study site.

In adjusted analyses, the discrimination of the secondary outcome by the organ dysfunction scores ranged between AUC 0.73 (0.70–0.77) for PELOD-2 and AUC 0.76 (0.73–0.80) for pSOFA (**eTable 4** and **Fig. S7*B***, http://links.lww.com/PCC/C434). Model calibration and discrimination were comparable for PELOD-2, pSOFA, and PODIUM in their binarized form (eTable 4 and **Fig. S8*B***, http://links.lww.com/PCC/C434). Results were confirmed in sensitivity analyses (**eTables 5** and **6**, http://links.lww.com/PCC/C434).

### Contribution of Individual Organ Dysfunctions to Mortality

**Figure 4.**
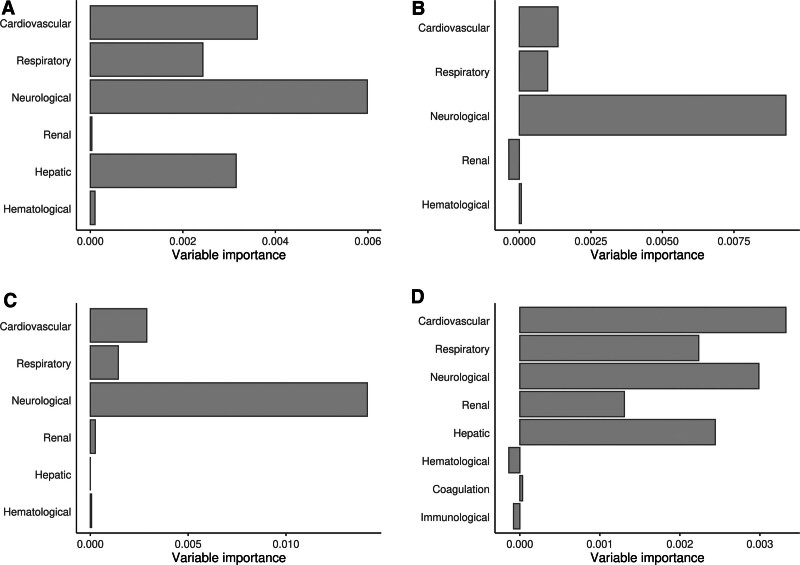
Importance of individual organ dysfunctions for the prediction of the primary outcome (30-d mortality) for each of the four organ dysfunction scores used. Permutation importance of individual organ dysfunctions from conditional random forest analyses of 2005 International Pediatric Sepsis Consensus Conference (**A**), Pediatric Logistic Organ Dysfunction-2 (**B**), pediatric Sequential Organ Failure Assessment (**C**), and Pediatric Organ Dysfunction Information Update Mandate (**D**). A higher value indicates higher importance compared with the other score items. Irrelevant covariates display permutation importance close to 0 or negative values.

We then assessed importance of individual organ dysfunctions as defined by IPSCC, PELOD-2, pSOFA, and PODIUM for the primary outcome using conditional random forest analyses. Cardiovascular, respiratory, and neurologic dysfunction were consistently the most relevant organ dysfunctions across all four organ dysfunction scores, while hepatic dysfunction was relevant in IPSCC and PODIUM but not pSOFA (**Fig. 4**). These results were confirmed in logic regression for IPSCC and binarized PELOD-2, while respiratory, neurologic, and hepatic dysfunction were most relevant for the binarized pSOFA. We could not derive a discriminative model from the binarized PODIUM using logic regression. Results were confirmed in sensitivity analyses (**Fig. S9**, http://links.lww.com/PCC/C434).

We then simplified IPSCC, PELOD-2, pSOFA, and PODIUM by only considering information on cardiovascular, respiratory, and neurologic dysfunction (**eTable 7**, http://links.lww.com/PCC/C434). In adjusted analyses, model calibration and discrimination were comparable to their full score equivalents (**eTables 8** and **9** and **Figs. S10** and **S11**, http://links.lww.com/PCC/C434). Results were confirmed in sensitivity analyses (**eTables** 7, **10**, and **11**, http://links.lww.com/PCC/C434).

## DISCUSSION

This secondary analysis of a curated dataset from 2011 to 2015 population-based multicenter prospective cohort study of children with blood culture-proven sepsis recruited across ED, ward, and PICU settings confirms an association between organ dysfunction and 30-day mortality. When comparing four pediatric scores for organ dysfunction, discrimination of the primary and secondary outcome did not substantially differ, however, we observed major differences in terms of classification as to whether organ dysfunction was present, and as to which organ was dysfunctional. Cardiovascular, respiratory, and neurologic dysfunction emerged as the most relevant organs contributing to a higher risk of mortality.

The relevance of organ dysfunction to identify children with higher disease severity is biologically evident and has been confirmed in multiple studies primarily in intensive care settings. Yet, direct comparisons of different approaches to adjudicate organ dysfunction, and understanding the impact of differences between scores, have received less attention in critically ill children (9). The Sepsis-3 criteria in adults give all organ dysfunctions similar weight and operationalize organ dysfunction by the SOFA score (4). In contrast, the IPSCC “severe sepsis” criteria ranked cardiovascular and respiratory dysfunction higher than other organ dysfunctions and provided specific criteria for individual organ dysfunction rather than using a standardized score (2). While the original purpose was to delineate children with severe sepsis to be enrolled in an interventional trial from children with sepsis without organ dysfunction, these criteria have been widely used amongst children with sepsis. The IPSCC was formulated by a panel of experts and has never undergone revision nor independent validation (3, 33). The Pediatric Sepsis Definition Taskforce is expected to develop and validate new criteria for pediatric sepsis (6, 15).

Interestingly, the variation in performance across the scores was only modest, despite substantial differences in how these scores were developed and constructed. PELOD-2 was derived from a French-Belgian multicenter study including 3671 children admitted to PICU between 2006 and 2007, and used weighting based on the effect size observed in the model (21). In comparison, the pSOFA was created combining the structured incremental SOFA score widely used in adults with PELOD-2 based age-specific cutoffs and has been subsequently validated in additional studies (14, 22). Finally, the recent PODIUM criteria represent the product of extensive systematic literature reviews and expert opinion, incorporating some age-specific cutoffs derived from other studies (such as creatinine) (9, 23). Importantly, the use of these scores, except for IPSCC, has been largely confined to PICU settings, and validation, where available, was almost exclusively performed in PICU cohorts (15, 34). To date, the majority of ED and ward-based studies investigating identification of sicker children have focused on Early Warning Tools, which commonly use vital signs but only rarely provide measures of organ dysfunction (35, 36).

Similar to the adult quick SOFA score (37), we found that cardiovascular, respiratory, and neurologic dysfunction were the most relevant organ dysfunctions in respect to prediction of the primary outcome across all four organ dysfunction scores. Our observation is supported by a post hoc analysis of a large randomized fluid trial on children with infection (38), and by a secondary analysis of an international pediatric sepsis point prevalence study (39). Interestingly, when we limited organ dysfunction scores to these three organ dysfunctions, the performance in unadjusted and adjusted analyses were surprisingly similar in comparison to the respective full organ dysfunction score. Importantly, cardiovascular, respiratory, and neurologic dysfunctions are readily measurable during clinical examination, which facilitates their assessment in ED and in smaller hospitals without dedicated PICU or in less resourced healthcare settings (6). However, our findings do not preclude that the presence of other organ dysfunction, such as renal failure, in particular, in higher acuity PICU cohorts, may contribute additionally to worse prognosis for short- and long-term outcomes (40).

In contrast to previous epidemiological studies on organ dysfunction in pediatric sepsis, the present study was based on data from a population-based multicenter prospective cohort designed to investigate blood culture-proven sepsis, with all patients meeting IPSCC sepsis criteria. The study captured both community- and hospital-acquired sepsis and is representative of a broad range of comorbidities and clinical settings in a high-income country (11–13). Sample size in subgroups was small, however, precluding separate analysis of PICU and ED populations. In addition, the focus on blood culture-proven sepsis allowed studying a clearly defined infectious disease phenotype of high relevance.

A number of limitations need to be considered. First, we excluded premature babies and those with early-onset sepsis, given the intrinsic difficulties in applying organ dysfunction criteria to this age group (41). Additionally, patients after allogeneic bone marrow transplants were not included. Second, we did not develop or validate specific thresholds but used published organ dysfunction criteria. Third, not all criteria included in the full PODIUM score were available, which may have decreased its performance. Fourth, we did not consider deaths occurring greater than 30 days, nor functional or quality of health outcomes (42). Fifth, the study only included children with blood culture-proven sepsis and our findings may not generalize to sepsis caused by viral infections or culture-negative sepsis. Finally, our findings date back to a cohort recruited in 2011–2015 and may not be generalizable to other healthcare settings, in particular, to low- or middle-income countries.

## CONCLUSIONS

In conclusion, while the performance of different organ dysfunction scores was similar to predict 30-day mortality, the substantial discrepancies in terms of organ dysfunction adjudication implies a lack of comparability and provides a strong rationale for future tailoring of these scores toward a single score informed by the analysis of large international databases (6, 16). Our findings suggest that derived, simplified organ dysfunction criteria may yield comparable performance, facilitating application toward less resourced and nonintensive care settings.

## ACKNOWLEDGMENTS

We thank the participating patients and their families. We thank the study nurses at each study site, and SwissPedNet, the Swiss Research Network of Clinical Pediatric Hubs, for their help in running the study. We also wish to acknowledge Torsten Hothorn for his valuable statistical input. Members of Study Group Swiss Pediatric Sepsis Study include (in alphabetical order): Christoph Aebi, Philipp K. A. Agyeman, Walter Bär, Christoph Berger, Sara Bernhard-Stirnemann, Eric Giannoni, Paul Hasters, Ulrich Heininger, Christian R. Kahlert, Gabriel Konetzny, Antonio Leone, Giancarlo Natalucci, Anita Niederer-Loher, Klara M. Posfay-Barbe, Christa Relly, Thomas Riedel, Luregn J. Schlapbach, and Martin Stocker.

## Supplementary Material


