# Supplementary material for: Organ Dysfunction in Children With Blood Culture-Proven Sepsis: Comparative Performance of Four Scores in a National Cohort Study
Source: Pediatr Crit Care Med. 2023 Oct 25;25(3):e117–28. doi: 10.1097/PCC.0000000000003388 (PMC10904004; doi:10.1097/PCC.0000000000003388)

Supplemental Digital Content

Organ Dysfunction in Children with Blood Culture-Proven Sepsis: Comparative Performance of Four Scores in a National Cohort Study

Luregn J Schlapbach MD, PhD, FCICM[1,2,3*], Sabrina Goertz MD[4*], Niels Hagenbuch MD, Msc[5], Blandine Aubert MD[6], Sebastien Papis MD[7], Eric Giannoni MD[6], Klara M Posfay-Barbe MD[7], Martin Stocker MD, MME[8], Ulrich Heininger MD[9], Sara Bernhard-Stirnemann MD, Msc[10], Anita Niederer-Loher MD[11], Christian R Kahlert MD[11], Giancarlo Natalucci MD[12], Christa Relly MD[4], Thomas Riedel MD[5,13], Christoph Aebi MD[5], Christoph Berger MD[4*], and Philipp KA Agyeman MD[5*] for the Swiss Pediatric Sepsis Study Group

*contributed equally

Table of contents

Supplementary Methods 4

eTable 1: Demographic and clinical characteristics of children with blood culture-proven sepsis 9

eTable 2: Proportions of patients with specific organ dysfunctions depending on the categorization by four organ dysfunction scores 11

eTable 3: Discrimination of the primary (30-day mortality) or secondary (30-day mortality and/or intensive care stay >= 3 days) outcome by organ dysfunction scores in children with sepsis 12

eTable 4: Measures of calibration and discrimination of adjusted models to predict the secondary outcome (30-day mortality and/or intensive care stay >= 3 days) 13

eTable 5: Measures of calibration and discrimination of adjusted models to predict the primary outcome (30-day mortality), when only considering the first sepsis episode in each patient 14

eTable 6: Measures of calibration and discrimination of adjusted models to predict the secondary outcome (30-day mortality and/or intensive care stay >= 3 days), when only considering the first sepsis episode in each patient 15

eTable 7: Discrimination of the primary (30-day mortality) or secondary (30-day mortality and/or intensive care stay >= 3 days) outcome by “simplified” organ dysfunction scores in children with sepsis 16

eTable 8: Measures of calibration and discrimination of adjusted models to predict the primary outcome (30-day mortality) with “simplified” organ dysfunction scores in children with sepsis 17

eTable 9: Measures of calibration and discrimination of adjusted models to predict the secondary outcome (30-day mortality and/or intensive care stay >= 3 days) with “simplified” organ dysfunction scores in children with sepsis 18

eTable 10: Measures of calibration and discrimination of adjusted models to predict the primary outcome (30-day mortality) with “simplified” organ dysfunction scores in children with sepsis, when only considering the first sepsis episode in each patient 19

eTable 11: Measures of calibration and discrimination of adjusted models to predict the secondary outcome (30-day mortality and/or intensive care stay >= 3 days) with “simplified” organ dysfunction scores in children with sepsis, when only considering the first sepsis episode in each patient 20

Figure S1: Consort Diagram on patient selection 21

Figure S2: Upsets demonstrating adjudication of organs affected by each of the four organ dysfunction scores 22

Figure S3: Proportion of episodes meeting the primary outcome in relation to the number of organs affected 23

Figure S4: Proportion of episodes meeting the secondary outcome in relation to the organ dysfunction score value 24

Figure S5: Proportions of episodes meeting the secondary outcome in relation to the number of organs affected 25

Figure S6: Receiver operating characteristics of the prediction of the primary outcome based on "binarized" organ dysfunction scores 26

Figure S7: Receiver operating characteristics of the prediction of the secondary outcome based on organ dysfunction scores 27

Figure S8: Receiver operating characteristics of the prediction of the secondary outcome based on "binarized" organ dysfunction scores 28

Figure S9: Importance of individual organ dysfunctions for the prediction of the primary outcome using only the first episode in each patient 29

Figure S10: Receiver operating characteristics of the prediction of the primary outcome based on "simplified" organ dysfunction scores 30

Figure S11: Receiver operating characteristics of the prediction of the secondary outcome based on "simplified" organ dysfunction scores 31

Supplementary Methods

Inclusion criteria for the Swiss Pediatric Sepsis Study

Neonates and children <17 years of age with blood culture-proven bacterial infection and systemic inflammatory response syndrome (SIRS), as defined by 2005 International Pediatric Sepsis Consensus Conference (IPSCC) (1), were eligible if they presented between September 1st, 2011, to December 31st, 2015 to one of the ten children's hospitals participating in the study. Patients were recruited consecutively and study data were prospectively recorded by medical study site investigators supported by local study nurses.

SIRS criteria used in the Swiss Pediatric Sepsis Study (based on 2005 IPSCC (1)):

For children beyond the neonatal period, SIRS was defined as the presence of at least two of the following four criteria, one of which had to be abnormal temperature or leukocyte count:

- body temperature <36°C or >38.5°C.
- abnormal heart rate for age in the absence of external stimuli, drugs, or congential heart disease.
- abnormal respiratory rate for age.
- Leukocyte count elevated or depressed for age (not secondary to chemotherapy-induced leukopenia) or >10% immature neutrophils.

Age specific limits for heart rate, respiratory rate, and leukocyte count were applied as defined in the 2005 IPSCC (1).

For neonates (<28 days old, or <44 weeks postconceptional age in premature newborns), at least two of the following signs were required to be present:

- tachycardia > 180/min.
- tachypnea >60/min or increased apnea frequency.
- temperature instability
- leukocyte count <5 or >34 x $10^3/mm^3$ or immature:total neutrophil ratio >0.2.
- capillary refill >2 seconds.
- apathia or irritability.

Exclusion criteria for the Swiss Pediatric Sepsis Study

Children with contaminated blood cultures, defined by the following criteria:

- coagulase-negative staphylococci in the absence of a central line at the time the blood culture was taken.
- blood cultures growing a mixed flora of different coagulase-negative staphylococci.
- blood cultures growing pathogens considered as contaminants by the physician in charge.

Children after allogeneic bone marrow transplantation

Score construction

International Pediatric Sepsis Consensus Conference (IPSCC) (1), Pediatric Logistic Organ Dysfunction-2 (PELOD-2) (2), pediatric Sequential Organ Failure Assessment (pSOFA) (3), and Pediatric Organ Dysfunction Information Update Mandate (PODIUM) scores (4) were calculated according to the originally published manuscripts. Specifically, if routine clinical data (such as heart rate, respiratory rate, blood pressure, etc.) for a patient and day were available, but organ-specific data such as creatinine values were missing, we assumed the value to be normal for that day.

For IPSCC, organ dysfunction was considered by each of six organs as per the original criteria for “severe sepsis”, except that we did not give cardiovascular or respiratory dysfunction a higher weight compared to other organs. Not all variables required to perform the full PODIUM score were available as they had not been routinely collected. Specifically, no data were available on Cornell Assessment of Pediatric Delirium (CAPD), electroencephalography, oxygenation index, troponin, central venous saturations, echocardiography, cardiac arrest, fibrinogen, D-dimers, hemoglobin, urine output, fluid overload, blood glucose, serum hormone levels, immunologic testing, and variables on gastrointestinal dysfunction. The PODIUM score was therefore calculated based on the available data for eight out of ten organs, not including endocrine dysfunction and gastrointestinal dysfunction. Organ-specific dysfunction was defined if the score summary of all variables contributing to a score value for that given organ were greater than zero. The table below shows for each organ dysfunction and score which variables were available.

Organ	Variable	N missing (%)	IPSCC	PELOD-2	pSOFA	PODIUM
Cardiovascular	Systolic blood pressure	123 (14%)	X			X
	Mean arterial blood pressure	128 (15%)		X	X	
	Inotrope/vasopressor	12 (1%)	X		X	X
	Lactate	432 (49%)	X	X		X
	Venoarterial ECMO	17 (2%)				X
	Heart rate	26 (3%)				X
	≥40ml fluid bolus given in 1 hour	17 (2%)	X			
Respiratory	Invasive ventilation	16 (2%)	X	X	X	X
	Non-invasive ventilation	17 (2%)	X		X	X
	pCO_2	761 (87%)	X	X		
	PaO_2	768 (88%)	X	X	X	X
	SpO_2	70 (8%)				X
	FiO_2	690 (79%)	X	X	X	X
	Venovenous or venoarterial ECMO	17 (2%)	X			X
Neurological	Glasgow Coma Scale	400 (46%)	X	X	X	X
	Pupillary reaction	311 (35%)		X		
Renal	Creatinine	391 (45%)	X	X	X	X
	Renal replacement therapy	24 (3%)				X
Hepatic	Bilirubin	630 (72%)	X		X	X
	Alanine transaminase	574 (65%)	X			X
	Glasgow Coma Scale	400 (46%)				X
Hematological	Platelets	62 (7%)	X	X	X	X
	INR	654 (75%)	X			
	Leukocyte count	54 (6%)		X		X
Immunological	Neutrophil count	163 (19%)				X
	Lymphocyte count	162 (18%)				X
Coagulation	Platelet	62 (7%)				X
	INR	654 (75%)				X

Statistical analysis

In the analysis of the 877 episodes from 807 patients, we considered episodes occurring in the same patient to be independent if the blood cultures defining sepsis onset were taken at least seven days apart. If more than one sepsis episode occurred in the last 30 days before a patient succumbed to his disease, we only considered the first sepsis episode for analysis.

Area Under the Receiver Operation Characteristics Curve Analysis

The discriminative power of the scores with respect to the primary and secondary outcome was assessed by the area under the curve (AUC) of receiver operation characteristics curves (ROC), calculated directly from the data. 95% confidence intervals (CIs) were estimated using DeLong's method (5), implemented in the R package pROC (version 1.18.0) (6).

To adjust the estimation of the ROC curves of the different scores for age (in years), sex (female, male), and presence of chronic medical conditions (healthy, comorbid), the scores were incorporated into a baseline logistic mixed-effects model with a random intercept for each hospital, using the function glmer from the package lme4 (version 1.1-27.1) (7). Based on the linear predictor of the fixed effects, ROC curves and corresponding AUCs with 95% CIs were calculated. The AUCs of the baseline model for the primary and the secondary outcome were 0.67 (95%-CI 0.58-0.76) and 0.59 (95%-CI 0.52-0.67), respectively.

Conditional Random Forests

The importance of the single organs in PELOD-2, pSFOA, and PODIUM was examined by conditional random forests that are unbiased in the presence of variables with different number of levels (8). Such a differential in levels is the case in PELOD-2 with renal dysfunction which is classified with two levels, whereas cardiovascular or neurological dysfunction are rated on a range of eight values.

To assess the importance of the individual score items, conditional random forest analyses were run, and the permutation importance was plotted (9). In this measure, by randomly permuting one covariate, and leaving the other covariates unpermuted, its association with the outcome is broken. Given there is a correspondence between X and Y, the prediction accuracy will drop, and the ensuing difference can be used as a qualitative measure for variable importance. Irrelevant covariates will display permutation importance values that are close to zero or even negative (the prediction accuracy does not suffer from removing a weak or non-existing relation). These variable importances should be read qualitatively, the values are not of concern. An advantage of this method compared to univariate screening methods is that the impact of each covariate is determined individually but in the multivariable context of all the other predictors. For the analysis, we used the function cforest, implemented in the package party (version 1.3-9) (10, 11) with the primary and secondary outcome as target variable, and the respective score items (= organs) as covariates. A total of 5'000 trees (parameter 'ntree') were simulated for the random forests, with a random selection of two variables per split (parameter 'mtry'). Due to the rather small number of covariates, smaller trees were grown which leads to a better convergence rate (9). Accordingly, at least five observations (parameter 'minsplit') were required in a node in order for a split to be attempted, and a terminal node had to contain at least three observations (parameter 'minbucket').

The value of the test statistic that must be exceeded to implement a split was kept at its default value of zero (parameter 'mincriterion'), equivalent to no threshold being applied.

Since each classification tree in a random forest analysis is built on a bootstrap sample (i.e. sampling with replacement the same number of observations as are in the data set), some observations, the "out-of-bag

observations," are not included in the data on which a tree is fit, but can later serve as a built-in test sample for calculating the prediction accuracy of that tree.

Logic Regression

The IPSCC score contains only binary items (organ dysfunction present or not present) and was analyzed with logic regression models (12). This method attempts to find the optimal logic combination of items in a semi-random process called simulated annealing. A host of Boolean expressions are formulated in the form of logic trees, where in each iteration a knot (one of the two operators 'and' or 'or') and a leave (a binary variable in the form of true or false, here: organ dysfunction present or not) is either added, removed, or switched in its logic content (exchange of 'present' to 'not present', and vice versa). With a binary outcome like death, the resulting tree allows for statements like "if conditions X1 and X2 are present in a patient, or conditions X3 or X4 but not X5 are present, then the outcome is more likely to be death."

The process of constructing a tree is repeated with an increasing limit of maximum number of leaves or covariates to be considered, resulting in potentially more complex logic conditions. The final trees are assessed by a scoring function, which, in case of classification, is the misclassification rate (for more details, also for using logic regression with a continuous outcome, see (12)).

To avoid overfitting the data by maximally complex trees, the models with different number of leaves are evaluated by their performance in cross-validation, i.e. their capability to correctly predict observations in a test data set that was left out of the training data set. The best model is chosen based on the score (misclassification rate), and with respect to parsimony. Hence, a more complex model with more covariates that only slightly outperforms a smaller model with a simpler logic structure is not necessarily better. To identify a single optimal logic tree, 500'000 iterations of adding, removing or switching were used. Within this process, a new tree with a lower misclassification error rate was always accepted. If a new tree had a higher error rate, the probability to accept it notwithstanding was 0.995 in the first 100'000, 0.951 in the second 100'000 iterations, about 0.0067 in the second to last, and 1.93×10^{-22} in the last 100'000 iterations (cooling scheme).

This method lessens the problem of greedy algorithms that always choose the better tree or stop if no tree can be found, and, thus, never visit the correct tree which might be two steps away (instead of one). We used the R package LogicReg (version 1.6.4). All analyses were conducted with R version 4.1.2 (13).

Supplementary References

1. Goldstein B, Giroir B, Randolph A, et al.: International pediatric sepsis consensus conference: definitions for sepsis and organ dysfunction in pediatrics. *Pediatric Critical Care Medicine: A Journal of the Society of Critical Care Medicine and the World Federation of Pediatric Intensive and Critical Care Societies* 2005; 6:2–8
2. Leteurtre S, Duhamel A, Salleron J, et al.: PELOD-2: an update of the PEdiatric Logistic Organ Dysfunction score. *Critical Care Medicine* 2013; 41:1761–1773
3. Matics TJ, Sanchez-Pinto LN: Adaptation and Validation of a Pediatric Sequential Organ Failure Assessment Score and Evaluation of the Sepsis-3 Definitions in Critically Ill Children. *JAMA pediatrics* 2017; 171:e172352
4. Bembea MM, Agus M, Akcan-Arikan A, et al.: Pediatric Organ Dysfunction Information Update Mandate (PODIUM) Contemporary Organ Dysfunction Criteria: Executive Summary. *Pediatrics* 2022; 149:S1–S12
5. DeLong ER, DeLong DM, Clarke-Pearson DL: Comparing the areas under two or more correlated receiver operating characteristic curves: a nonparametric approach. *Biometrics* 1988; 44:837–845

6. Robin X, Turck N, Hainard A, et al.: pROC: an open-source package for R and S+ to analyze and compare ROC curves [Internet]. *BMC Bioinformatics* 2011; 12:77Available from: https://bmcbioinformatics.biomedcentral.com/articles/10.1186/1471-2105-12-77
7. Bates D, Mächler M, Bolker B, et al.: Fitting Linear Mixed-Effects Models Using lme4 [Internet]. *Journal of Statistical Software* 2015; 67Available from: http://www.jstatsoft.org/v67/i01/
8. Hothorn T, Hornik K, Zeileis A: Unbiased Recursive Partitioning: A Conditional Inference Framework [Internet]. *Journal of Computational and Graphical Statistics* 2006; 15:651–674 Available from: http://www.tandfonline.com/doi/abs/10.1198/106186006X133933
9. Strobl C, Malley J, Tutz G: An introduction to recursive partitioning: rationale, application, and characteristics of classification and regression trees, bagging, and random forests. *Psychological Methods* 2009; 14:323–348
10. Strobl C, Boulesteix A-L, Zeileis A, et al.: Bias in random forest variable importance measures: Illustrations, sources and a solution [Internet]. *BMC Bioinformatics* 2007; 8:25 Available from: https://bmcbioinformatics.biomedcentral.com/articles/10.1186/1471-2105-8-25
11. Strobl C, Boulesteix A-L, Kneib T, et al.: Conditional variable importance for random forests [Internet]. *BMC Bioinformatics* 2008; 9:307 Available from: https://bmcbioinformatics.biomedcentral.com/articles/10.1186/1471-2105-9-307
12. Ruczinski I, Kooperberg C, LeBlanc M: Logic Regression [Internet]. *Journal of Computational and Graphical Statistics* 2003; 12:475–511 Available from: http://www.tandfonline.com/doi/abs/10.1198/1061860032238
13. R Core Team: R: A language and environment for statistical computing [Internet]. Vienna, Austria: R Foundation for Statistical Computing; 2021. Available from: https://www.R-project.org/

eTable 1: Demographic and clinical characteristics of children with blood culture-proven sepsis

Categorical variables are presented as counts (percentages) and continuous variables as median (interquartile range). Column percentages are presented; percentages are based on available data for each variable. PICU, pediatric intensive care unit; LOS, length of stay.

	All sepsis episodes (n=877)	Sepsis episodes in previously healthy children (n=442)	Sepsis episodes in children with comorbidity (n=435)
Age at sepsis onset (months)	31.9 (4.6 - 94.3)	30.1 (2.4 - 88.2)	33.9 (7.4 - 95.7)
Male sex	520 (59%)	265 (60%)	255 (59%)
Comorbidities			
Neurological or neuromuscular	16 (2%)		16 (4%)
Cardiovascular	23 (3%)		23 (5%)
Respiratory	6 (1%)		6 (1%)
Renal and urological	23 (3%)		23 (5%)
Gastrointestinal	38 (4%)		38 (9%)
Hematological or immunological	12 (1%)		12 (3%)
Metabolic	4 (<1%)		4 (1%)
Other congenital or genetic defect	2 (<1%)		2 (<1%)
Malignant disease	137 (16%)		137 (31%)
Surgery or burn	26 (3%)		26 (6%)
Technology dependence	17 (2%)		17 (4%)
More than one condition	131 (15%)		131 (30%)
Hospital-acquired sepsis	227 (26%)	19 (4%)	208 (48%)
LOS after sepsis onset (days)	12 (7 - 21)	10 (7 - 14)	16 (10 - 36)
Pathogens			
S. aureus	136 (16%)	70 (16%)	66 (15%)
Coagulase-negative staphylococci	70 (8%)	3 (1%)	67 (15%)
S. pneumoniae	113 (13%)	90 (20%)	23 (5%)
Viridans group streptococci	48 (5%)	14 (3%)	34 (8%)
Group A streptococci	52 (6%)	49 (11%)	3 (1%)
Group B streptococci	47 (5%)	46 (10%)	1 (<1%)
Enterococcus spp	28 (3%)	6 (1%)	22 (5%)
Other Gram-positive bacteria	24 (3%)	12 (3%)	12 (3%)
E. coli	175 (20%)	90 (20%)	85 (20%)
H. influenzae	20 (2%)	17 (4%)	3 (1%)
Klebsiella spp	36 (4%)		36 (8%)
N. meningitidis	28 (3%)	26 (6%)	2 (<1%)
P. aeruginosa	20 (2%)	2 (<1%)	18 (4%)
Other Gram-negative bacteria	64 (7%)	17 (4%)	47 (11%)
C. albicans	16 (2%)		16 (4%)
Site or type of infection			
Primary bloodstream	132 (15%)	80 (18%)	52 (12%)

	All sepsis episodes (n=877)	Sepsis episodes in previously healthy children (n=442)	Sepsis episodes in children with comorbidity (n=435)
Central line-associated bloodstream	227 (26%)		227 (52%)
Urinary tract	113 (13%)	77 (17%)	36 (8%)
Pneumonia	90 (10%)	76 (17%)	14 (3%)
Central nervous system	64 (7%)	57 (13%)	7 (2%)
Bone and joints	67 (8%)	62 (14%)	5 (1%)
Gastrointestinal system	48 (5%)	19 (4%)	29 (7%)
Skin and soft tissue	44 (5%)	27 (6%)	17 (4%)
Surgical site	16 (2%)	1 (<1%)	15 (3%)
Ear, nose, and throat	24 (3%)	22 (5%)	2 (<1%)
Cardiovascular	13 (1%)	1 (<1%)	12 (3%)
Toxic shock syndrome	7 (1%)	5 (1%)	2 (<1%)
Other specific infection type	32 (4%)	15 (3%)	17 (4%)
PICU admission	289 (33%)	120 (27%)	169 (39%)
PICU LOS (days)	6 (2 - 15)	3 (1 - 9)	9 (3 - 32)
Case fatality	38 (4%)	10 (2%)	28 (6%)
Death or PICU LOS ≥ 3 days	226 (26%)	79 (18%)	147 (34%)

eTable 2: Proportions of patients with specific organ dysfunctions depending on the categorization by four organ dysfunction scores

Counts (percentages) are shown for children without organ dysfunction, as opposed to children with organ dysfunction, split into different organs depending on the score. Percent agreement and Krippendorff's alpha (both with 95% confidence intervals derived from 5000 bootstrap samples) are shown for absence of organ dysfunction and the presence of individual organ dysfunctions that are assessed by at least two different scores. IPSCC, International Pediatric Sepsis Consensus Conference; PELOD-2, Pediatric Logistic Organ Dysfunction-2; pSOFA, pediatric Sequential Organ Failure Assessment; PODIUM, Pediatric Organ Dysfunction Information Update Mandate

	IPSCC	PELOD-2	pSOFA	PODIUM	Agreement (95% CI)	Krippendorff's alpha (95% CI)
No organ dysfunction	590 (67.3%)	401 (45.7%)	392 (44.7%)	416 (47.4%)	57.4 (54 - 60.7)	0.53 (0.49 - 0.56)
Cardiovascular dysfunction	83 (9.5%)	260 (29.6%)	252 (28.7%)	202 (23.0%)	68.1 (65 - 71.2)	0.47 (0.43 - 0.52)
Respiratory dysfunction	70 (8.0%)	68 (7.8%)	79 (9.0%)	63 (7.2%)	94.2 (92.6 - 95.8)	0.79 (0.73 - 0.84)
Neurological dysfunction	65 (7.4%)	59 (6.7%)	113 (12.9%)	41 (4.7%)	91.2 (89.3 - 93)	0.68 (0.61 - 0.74)
Renal dysfunction	31 (3.5%)	139 (15.8%)	107 (12.2%)	50 (5.7%)	85.9 (83.6 - 88.1)	0.52 (0.46 - 0.58)
Hepatic dysfunction	58 (6.6%)		61 (7.0%)	10 (1.1%)	89.5 (87.5 - 91.4)	0.25 (0.16 - 0.34)
Hematological dysfunction	153 (17.4%)	242 (27.6%)	243 (27.7%)	200 (22.8%)	82.8 (80.2 - 85.3)	0.73 (0.68 - 0.76)
Coagulation dysfunction				21 (2.4%)		
Immunological dysfunction				221 (25.2%)		

eTable 3: Discrimination of the primary (30-day mortality) or secondary (30-day mortality and/or intensive care stay >= 3 days) outcome by organ dysfunction scores in children with sepsis

Area under the curve of receiver operating characteristics curves and their 95% confidence intervals are shown for the original scores and binarized PELOD-2, pSOFA, and PODIUM. IPSCC, International Pediatric Sepsis Consensus Conference; PELOD-2, Pediatric Logistic Organ Dysfunction-2; pSOFA, pediatric Sequential Organ Failure Assessment; PODIUM, Pediatric Organ Dysfunction Information Update Mandate.

Score		All sepsis episodes		First sepsis episode in each patient only	
		Primary outcome	Secondary outcome	Primary outcome	Secondary outcome
**Original Scores**	IPSCC	0.82 (0.74 - 0.90)	0.68 (0.64 - 0.72)	0.83 (0.75 - 0.91)	0.69 (0.65 - 0.73)
	PELOD-2	0.73 (0.63 - 0.83)	0.67 (0.62 - 0.71)	0.75 (0.64 - 0.86)	0.67 (0.63 - 0.72)
	pSOFA	0.78 (0.69 - 0.88)	0.71 (0.67 - 0.75)	0.78 (0.68 - 0.89)	0.71 (0.67 - 0.75)
	PODIUM	0.77 (0.68 - 0.87)	0.70 (0.66 - 0.74)	0.78 (0.68 - 0.89)	0.71 (0.67 - 0.75)
**Binarized–scores**	PELOD-2	0.73 (0.63 - 0.83)	0.67 (0.63 - 0.71)	0.75 (0.64 - 0.85)	0.67 (0.63 - 0.72)
	pSOFA	0.75 (0.65 - 0.84)	0.71 (0.67 - 0.75)	0.75 (0.65 - 0.85)	0.70 (0.66 - 0.74)
	PODIUM	0.77 (0.68 - 0.86)	0.70 (0.66 - 0.74)	0.79 (0.68 - 0.89)	0.71 (0.67 - 0.75)

eTable 4: Measures of calibration and discrimination of adjusted models to predict the secondary outcome (30-day mortality and/or intensive care stay >= 3 days)

Analyses adjusted for age, sex, and presence of comorbidities, with a random effect per study site are shown. AUC, Area under the curve; IPSCC, International Pediatric Sepsis Consensus Conference; PELOD-2, Pediatric Logistic Organ Dysfunction-2; pSOFA, pediatric Sequential Organ Failure Assessment; PODIUM, Pediatric Organ Dysfunction Information Update Mandate.

	Original scores				Binarized scores		
	**IPSCC**	**PELOD-2**	**pSOFA**	**PODIUM**	**PELOD-2**	**pSOFA**	**PODIUM**
AUC (95%-CI)	0.76 (0.72 – 0.80)	0.73 (0.70 - 0.77)	0.76 (0.73 - 0.80)	0.75 (0.72 - 0.79)	0.74 (0.70 - 0.78)	0.76 (0.73 - 0.80)	0.75 (0.71 - 0.79)
Hosmer-Lemeshow C*							
X^2 (8)	37.7	18.9	14.26	13.95	10.46	23.8	11.34
p value	<0.001	0.015	0.075	0.083	0.23	0.002	0.18
Cox's Calibration regression							
Intercept (95%-CI)	0.01 (-0.10 – 0.12)	0.02 (-0.07 – 0.10)	0.01 (-0.07 – 0.09)	0.00 (-0.08 – 0.06)	0.01 (-0.05 – 0.07)	0.01 (-0.08 – 0.10)	0.00 (-0.06 – 0.05)
Slope (95%-CI)	0.97 (0.62 – 1.32)	0.94 (0.67 – 1.20)	0.98 (0.74 – 1.22)	1.04 (0.82 – 1.26)	0.95 (0.77 – 1.13)	0.96 (0.68 – 1.23)	1.02 (0.85 – 1.20)
X^2 (1)	149	112	169	159	132	161	153
p value	<0.001	<0.001	<0.001	<0.001	<0.001	<0.001	<0.001
Brier's score	0.16	0.16	0.15	0.15	0.16	0.15	0.16

eTable 5: Measures of calibration and discrimination of adjusted models to predict the primary outcome (30-day mortality), when only considering the first sepsis episode in each patient

Analyses adjusted for age, sex, and presence of comorbidities, with a random effect per study site are shown. AUC, Area under the curve; IPSCC, International Pediatric Sepsis Consensus Conference; PELOD-2, Pediatric Logistic Organ Dysfunction-2; pSOFA, pediatric Sequential Organ Failure Assessment; PODIUM, Pediatric Organ Dysfunction Information Update Mandate.

	**Original scores**				**Binarized scores**		
	**IPSCC**	**PELOD-2**	**pSOFA**	**PODIUM**	**PELOD-2**	**pSOFA**	**PODIUM**
AUC (95%-CI)	0.89 (0.83 - 0.94)	0.85 (0.78 - 0.91)	0.86 (0.79 - 0.93)	0.85 (0.78 - 0.92)	0.83 (0.76 - 0.90)	0.82 (0.74 - 0.90)	0.85 (0.78 - 0.92)
Hosmer-Lemeshow C*							
X^2 (8)	5.8	13.31	6.9	17.28	15.55	9.7	35
p value	0.7	0.10	0.5	0.027	0.049	0.3	<0.001
Cox's Calibration regression							
Intercept (95%-CI)	0.00 (-0.01 – 0.01)	0.01 (-0.01 – 0.02)	0.00 (-0.01 – 0.01)	0.00 (-0.02 – 0.02)	0.00 (-0.02 – 0.02)	0.00 (-0.01 – 0.02)	0.00 (-0.02 – 0.03)
Slope (95%-CI)	1.01 (0.88 – 1.14)	0.87 (0.68 1.07)	1.00 (0.84 – 1.16)	1.01 (0.75 – 1.28)	1.00 (0.71 – 1.28)	0.99 (0.80 – 1.19)	0.98 (0.68 – 1.28)
X^2 (1)	65	47	63	63	51	45	57
p value	<0.001	<0.001	<0.001	<0.001	<0.001	<0.001	<0.001
Brier's score	0.03	0.03	0.03	0.03	0.03	0.03	0.03

eTable 6: Measures of calibration and discrimination of adjusted models to predict the secondary outcome (30-day mortality and/or intensive care stay >= 3 days), when only considering the first sepsis episode in each patient

Analyses adjusted for age, sex, and presence of comorbidities, with a random effect per study site are shown. AUC, Area under the curve; IPSCC, International Pediatric Sepsis Consensus Conference; PELOD-2, Pediatric Logistic Organ Dysfunction-2; pSOFA, pediatric Sequential Organ Failure Assessment; PODIUM, Pediatric Organ Dysfunction Information Update Mandate.

	**Original scores**				**Binarized scores**		
	**IPSCC**	**PELOD-2**	**pSOFA**	**PODIUM**	**PELOD-2**	**pSOFA**	**PODIUM**
AUC (95%-CI)	0.77 (0.73 - 0.81)	0.75 (0.71 - 0.78)	0.77 (0.74 - 0.81)	0.76 (0.72 - 0.80)	0.75 (0.71 - 0.79)	0.77 (0.73 - 0.81)	0.76 (0.72 - 0.80)
Hosmer-Lemeshow C*							
X^2 (8)	34	23.2	16.4	18.3	20.8	18	16
p value	<0.001	0.003	0.037	0.019	0.008	0.021	0.043
Cox's Calibration regression							
Intercept (95%-CI)	0.01 (-0.10 – 0.13)	0.02 (-0.07 – 0.12)	0.02 (-0.06 – 0.10)	0.00 (-0.07 – 0.08)	0.02 (-0.06 – 0.09)	0.01 (-0.07 – 0.10)	0.00 (-0.07 – 0.07)
Slope (95%-CI)	0.96 (0.62 – 1.31)	0.91 (0.61 – 1.21)	0.94 (0.71 – 1.18)	0.99 (0.76 – 1.23)	0.93 (0.69 – 1.16)	0.96 (0.71 – 1.22)	1.00 (0.80 – 1.21)
X^2 (1)	149	107	154	146	125	156	149
p value	<0.001	<0.001	<0.001	<0.001	<0.001	<0.001	<0.001
Brier's score	0.15	0.16	0.15	0.15	0.15	0.15	0.15

eTable 7: Discrimination of the primary (30-day mortality) or secondary (30-day mortality and/or intensive care stay >= 3 days) outcome by "simplified" organ dysfunction scores in children with sepsis

Simplified organ dysfunction scores were constructed only considering cardiovascular, respiratory, and neurological dysfunction. Area under the curve of receiver operating characteristics curves and their 95% confidence intervals are shown for the original scores and binarized PELOD-2, pSOFA, and PODIUM. IPSCC, International Pediatric Sepsis Consensus Conference; PELOD-2, Pediatric Logistic Organ Dysfunction-2; pSOFA, pediatric Sequential Organ Failure Assessment; PODIUM, Pediatric Organ Dysfunction Information Update Mandate.

Score		All episodes		First sepsis episode in each patient only	
		Primary outcome	Secondary outcome	Primary outcome	Secondary outcome
Original Scores	IPSCC	0.78 (0.70 - 0.87)	0.69 (0.66 – 0.73)	0.80 (0.71 - 0.88)	0.70 (0.66 – 0.73)
	PELOD-2	0.72 (0.62 – 0.82)	0.70 (0.66 – 0.74)	0.74 (0.63 – 0.84)	0.70 (0.66 – 0.74)
	pSOFA	0.76 (0.66 – 0.86)	0.75 (0.71 – 0.79)	0.76 (0.66 – 0.86)	0.74 (0.71 – 0.78)
	PODIUM	0.77 (0.68 – 0.86)	0.73 (0.69 – 0.77)	0.77 (0.68 – 0.87)	0.73 (0.69 – 0.76)
Binarized-Scores	PELOD-2	0.72 (0.63 – 0.82)	0.69 (0.65 – 0.73)	0.74 (0.64 – 0.84)	0.69 (0.65 – 0.73)
	pSOFA	0.73 (0.64 – 0.83)	0.73 (0.70 – 0.77)	0.74 (0.64 – 0.84)	0.73 (0.69 – 0.77)
	PODIUM	0.77 (0.68 – 0.86)	0.73 (0.70 – 0.77)	0.77 (0.68 – 0.87)	0.73 (0.69 – 0.77)

eTable 8: Measures of calibration and discrimination of adjusted models to predict the primary outcome (30-day mortality) with "simplified" organ dysfunction scores in children with sepsis

Simplified organ dysfunction scores were constructed only considering cardiovascular, respiratory, and neurological dysfunction. Analyses adjusted for age, sex, and presence of comorbidities, with a random effect per study site are shown. AUC, Area under the curve; IPSCC, International Pediatric Sepsis Consensus Conference; PELOD-2, Pediatric Logistic Organ Dysfunction-2; pSOFA, pediatric Sequential Organ Failure Assessment; PODIUM, Pediatric Organ Dysfunction Information Update Mandate.

	Original scores				Binarized scores		
	**IPSCC**	**PELOD-2**	**pSOFA**	**PODIUM**	**PELOD-2**	**pSOFA**	**PODIUM**
AUC (95%-CI)	0.89 (0.84 – 0.94)	0.85 (0.79 – 0.91)	0.87 (0.81 – 0.93)	0.88 (0.83 – 0.93)	0.84 (0.78 – 0.89)	0.83 (0.76 – 0.89)	0.88 (0.84 – 0.93)
Hosmer-Lemeshow C*							
X^2 (8)	6.1	23.7	5.8	4.1	13.4	9.6	5.0
p value	0.6	0.003	0.7	0.8	0.099	0.3	0.8
Cox's Calibration regression							
Intercept (95%-CI)	0.00 (-0.01 – 0.02)	0.01 (-0.02 – 0.04)	0.00 (-0.01 – 0.01)	0.00 (-0.01 – 0.01)	0.01 (-0.01 – 0.03)	0.00 (-0.01 – 0.02)	0.00 (-0.01 – 0.01)
Slope (95%-CI)	0.90 (0.76 – 1.04)	0.84 (0.50 – 1.18)	0.90 (0.79 – 1.01)	0.97 (0.83 – 1.11)	0.85 (0.60 – 1.10)	0.93 (0.74 – 1.12)	1.02 (0.85 – 1.18)
X^2 (1)	64	44	59	69	40	46	69
p value	<0.001	<0.001	<0.001	<0.001	<0.001	<0.001	<0.001
Brier's score	0.03	0.03	0.03	0.03	0.03	0.03	0.03

eTable 9: Measures of calibration and discrimination of adjusted models to predict the secondary outcome (30-day mortality and/or intensive care stay >= 3 days) with "simplified" organ dysfunction scores in children with sepsis

Simplified organ dysfunction scores were constructed only considering cardiovascular, respiratory, and neurological dysfunction. Analyses adjusted for age, sex, and presence of comorbidities, with a random effect per study site are shown. AUC, Area under the curve; IPSCC, International Pediatric Sepsis Consensus Conference; PELOD-2, Pediatric Logistic Organ Dysfunction-2; pSOFA, pediatric Sequential Organ Failure Assessment; PODIUM, Pediatric Organ Dysfunction Information Update Mandate.

	Original scores				Binarized scores		
	**IPSCC**	**PELOD-2**	**pSOFA**	**PODIUM**	**PELOD-2**	**pSOFA**	**PODIUM**
AUC (95%-CI)	0.81 (0.78 – 0.85)	0.78 (0.74 – 0.81)	0.82 (0.78 – 0.85)	0.79 (0.76 – 0.83)	0.77 (0.74 – 0.81)	0.81 (0.77 – 0.84)	0.80 (0.76 – 0.83)
Hosmer-Lemeshow C*							
X^2 (8)	16.2	14.7	11	12.7	21.8	26.2	9.8
p value	0.04	0.06	0.2	0.12	0.005	0.001	0.3
Cox's Calibration regression							
Intercept (95%-CI)	0.00 (-0.08 – 0.08)	0.00 (-0.08 – 0.08)	0.00 (-0.05 – 0.06)	0.01 (-0.06 – 0.07)	0.00 (-0.09 – 0.10)	0.01 (-0.07 – 0.09)	0.00 (-0.05 – 0.05)
Slope (95%-CI)	1.01 (0.78 – 1.23)	1.01 (0.77 – 1.25)	0.99 (0.83 – 1.14)"	0.99 (0.79 – 1.18)	1.00 (0.70 – 1.29)	0.97 (0.73 – 1.21)	1.02 (0.87 – 1.17)
X^2 (1)	231	180	173	249	208	194	223
p value	<0.001	<0.001	<0.001	<0.001	<0.001	<0.001	<0.001
Brier's score	0.14	0.15	0.13	0.14	0.15	0.14	0.14

eTable 10: Measures of calibration and discrimination of adjusted models to predict the primary outcome (30-day mortality) with "simplified" organ dysfunction scores in children with sepsis, when only considering the first sepsis episode in each patient

Simplified organ dysfunction scores were constructed only considering cardiovascular, respiratory, and neurological dysfunction. Analyses adjusted for age, sex, and presence of comorbidities, with a random effect per study site are shown. AUC, Area under the curve; IPSCC, International Pediatric Sepsis Consensus Conference; PELOD-2, Pediatric Logistic Organ Dysfunction-2; pSOFA, pediatric Sequential Organ Failure Assessment; PODIUM, Pediatric Organ Dysfunction Information Update Mandate.

	Original scores				Binarized scores		
	**IPSCC**	**PELOD-2**	**pSOFA**	**PODIUM**	**PELOD-2**	**pSOFA**	**PODIUM**
AUC (95%-CI)	0.89 (0.84 – 0.95)	0.86 (0.80 – 0.92)	0.86 (0.80 – 0.93)	0.88 (0.83 – 0.94)	0.84 (0.78 – 0.90)	0.83 (0.76 – 0.90)	0.88 (0.84 – 0.93)
Hosmer-Lemeshow C*							
X^2 (8)	7.4	9.9	5.3	4.6	11.9	11.5	7.2
p value	0.5	0.3	0.7	0.8	0.15	0.18	0.5
Cox's Calibration regression							
Intercept (95%-CI)	0.00 (-0.01 – 0.02)	0.01 (-0.01 – 0.03)	0.01 (-0.00 – 0.01)	0.00 (-0.00 – 0.01)	0.01 (-0.01 – 0.03)	0.00 (-0.02 – 0.02)	0.00 (-0.01 – 0.01)
Slope (95%-CI)	0.92 (0.78 – 1.06)	0.83 (0.59 – 1.07)	0.86 (0.77 – 0.95)	0.92 (0.83 – 1.02)	0.83 (0.60 – 1.05)	0.95 (0.70 – 1.21)	0.95 (0.81 – 1.08)
X^2 (1)	64	44	53	59	38	48	59
p value	<0.001	<0.001	<0.001	<0.001	<0.001	<0.001	<0.001
Brier's score	0.03	0.03	0.03	0.03	0.03	0.03	0.03

eTable 11: Measures of calibration and discrimination of adjusted models to predict the secondary outcome (30-day mortality and/or intensive care stay >= 3 days) with "simplified" organ dysfunction scores in children with sepsis, when only considering the first sepsis episode in each patient

Simplified organ dysfunction scores were constructed only considering cardiovascular, respiratory, and neurological dysfunction. Analyses adjusted for age, sex, and presence of comorbidities, with a random effect per study site are shown. AUC, Area under the curve; IPSCC, International Pediatric Sepsis Consensus Conference; PELOD-2, Pediatric Logistic Organ Dysfunction-2; pSOFA, pediatric Sequential Organ Failure Assessment; PODIUM, Pediatric Organ Dysfunction Information Update Mandate.

	**Original scores**				**Binarized scores**		
	**IPSCC**	**PELOD-2**	**pSOFA**	**PODIUM**	**PELOD-2**	**pSOFA**	**PODIUM**
AUC (95%-CI)	0.82 (0.78 – 0.85)	0.79 (0.75 – 0.82)	0.82 (0.78 – 0.85)	0.79 (0.76 – 0.83)	0.78 (0.74 – 0.82)	0.81 (0.77 – 0.84)	0.80 (0.76 – 0.84)
Hosmer-Lemeshow C*							
X^2 (8)	13.1	20.1	13.1	10.5	16.6	23.0	11.2
p value	0.11	0.010	0.11	0.2	0.035	0.003	0.2
Cox's Calibration regression							
Intercept (95%-CI)	0.00 (-0.08 – 0.07)	0.00 (-0.08 – 0.08)	0.00 (-0.06 – 0.06)	0.01 (-0.05 – 0.06)	0.01 (-0.08 – 0.09)	0.01 (-0.08 – 0.10)	0.00 (-0.06 – 0.05)
Slope (95%-CI)	1.01 (0.81 – 1.21)	1.01 (0.77 – 1.25)	1.00 (0.83 – 1.17)	0.99 (0.83 – 1.15)	0.98 (0.73 – 1.24)	0.95 (0.69 – 1.21)	1.03 (0.87 – 1.18)
X^2 (1)	227	117	243	187	163	187	215
p value	<0.001	<0.001	<0.001	<0.001	<0.001	<0.001	<0.001
Brier's score	0.14	0.15	0.13	0.14	0.15	0.14	0.14

Figure S1: Consort Diagram on patient selection

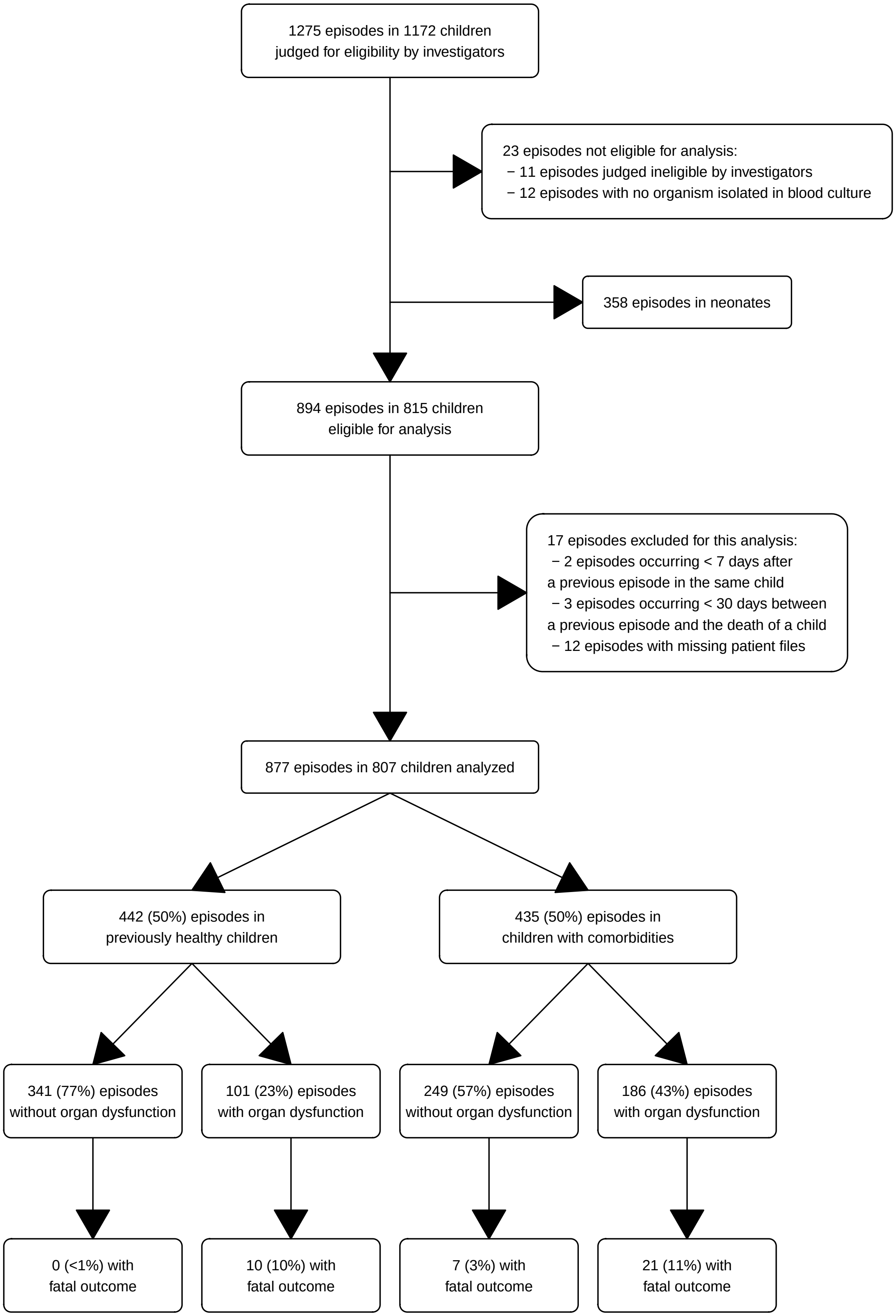

Figure S2: Upsets demonstrating adjudication of organs affected by each of the four organ dysfunction scores

UpSets displaying intersections of the four organ dysfunction scores (sets). The lower panel shows a matrix layout for all intersections of the sets. Dark circles indicate a sets is part of the intersection (i.e. an organ dysfunction was present according to the respective score). The upper panel shows the size of intersections as stacked bar chart with light fill representing children who survived and dark fill children who died. IPSCC, International Pediatric Sepsis Consensus Conference; PELOD-2, Pediatric Logistic Organ Dysfunction-2; pSOFA, pediatric Sequential Organ Failure Assessment; PODIUM, Pediatric Organ Dysfunction Information Update Mandate.

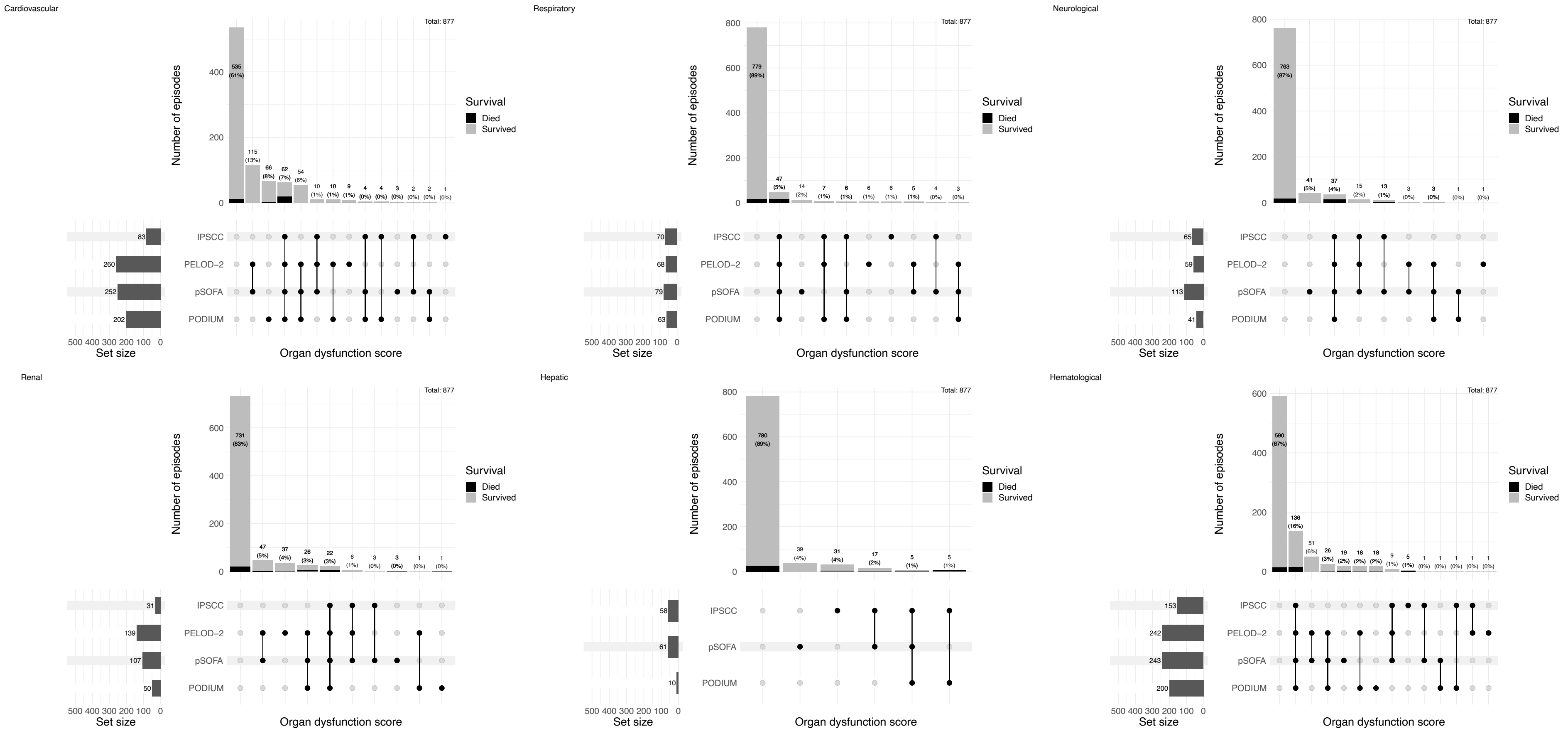

Figure S3: Proportion of episodes meeting the primary outcome in relation to the number of organs affected

IPSCC, International Pediatric Sepsis Consensus Conference; PELOD-2, Pediatric Logistic Organ Dysfunction-2; pSOFA, pediatric Sequential Organ Failure Assessment; PODIUM, Pediatric Organ Dysfunction Information Update Mandate

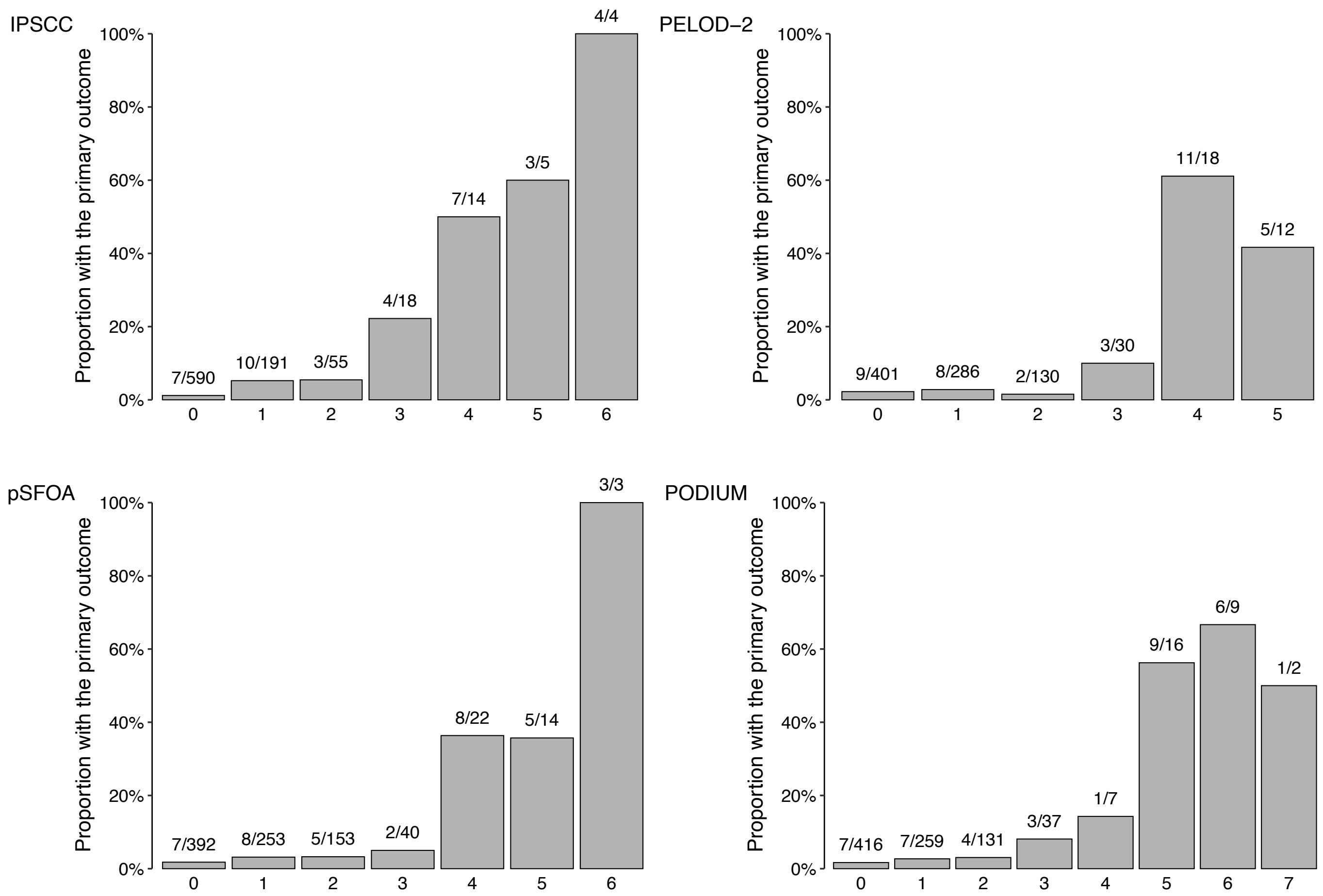

Figure S4: Proportion of episodes meeting the secondary outcome in relation to the organ dysfunction score value

IPSCC, International Pediatric Sepsis Consensus Conference; PELOD-2, Pediatric Logistic Organ Dysfunction-2; pSOFA, pediatric Sequential Organ Failure Assessment; PODIUM, Pediatric Organ Dysfunction Information Update Mandate

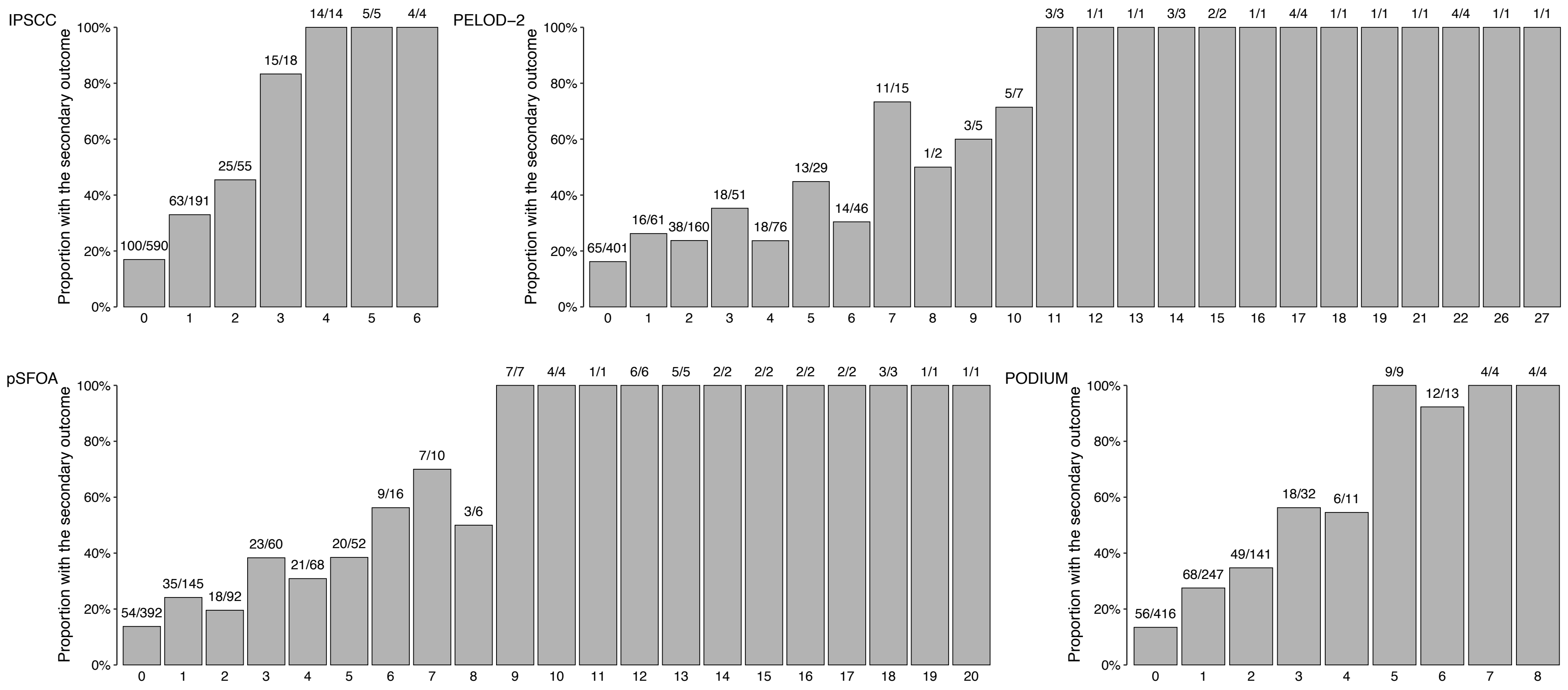

Figure S5: Proportions of episodes meeting the secondary outcome in relation to the number of organs affected

IPSCC, International Pediatric Sepsis Consensus Conference; PELOD-2, Pediatric Logistic Organ Dysfunction-2; pSOFA, pediatric Sequential Organ Failure Assessment; PODIUM, Pediatric Organ Dysfunction Information Update Mandate

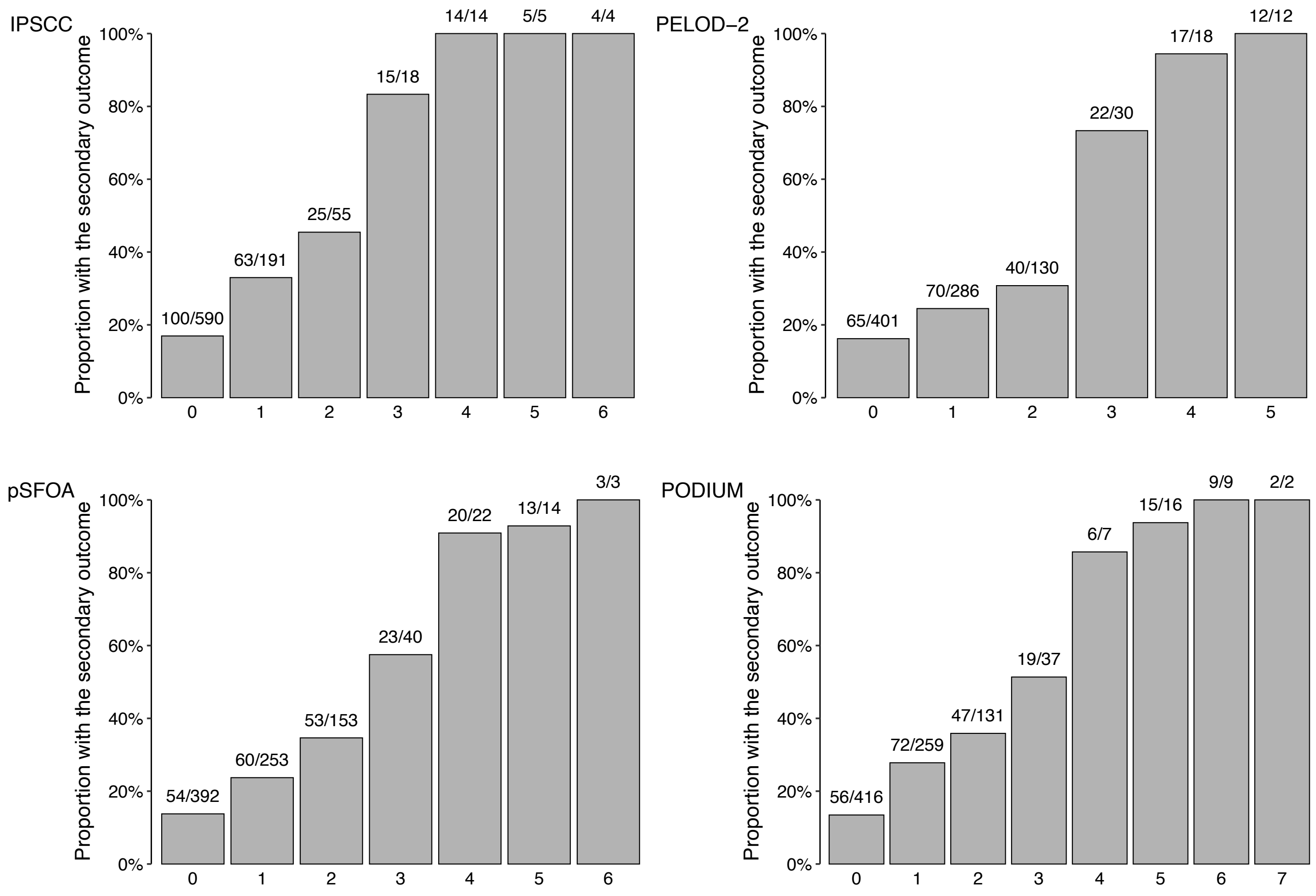

Figure S6: Receiver operating characteristics of the prediction of the primary outcome based on "binarized" organ dysfunction scores

Receiver operating characteristics curves based on unadjusted analyses (Panel A) and analyses adjusted for age (years), sex, and presence of a comorbidity (Panel B). IPSCC, International Pediatric Sepsis Consensus Conference; PELOD-2, Pediatric Logistic Organ Dysfunction-2; pSOFA, pediatric Sequential Organ Failure Assessment; PODIUM, Pediatric Organ Dysfunction Information Update Mandate.

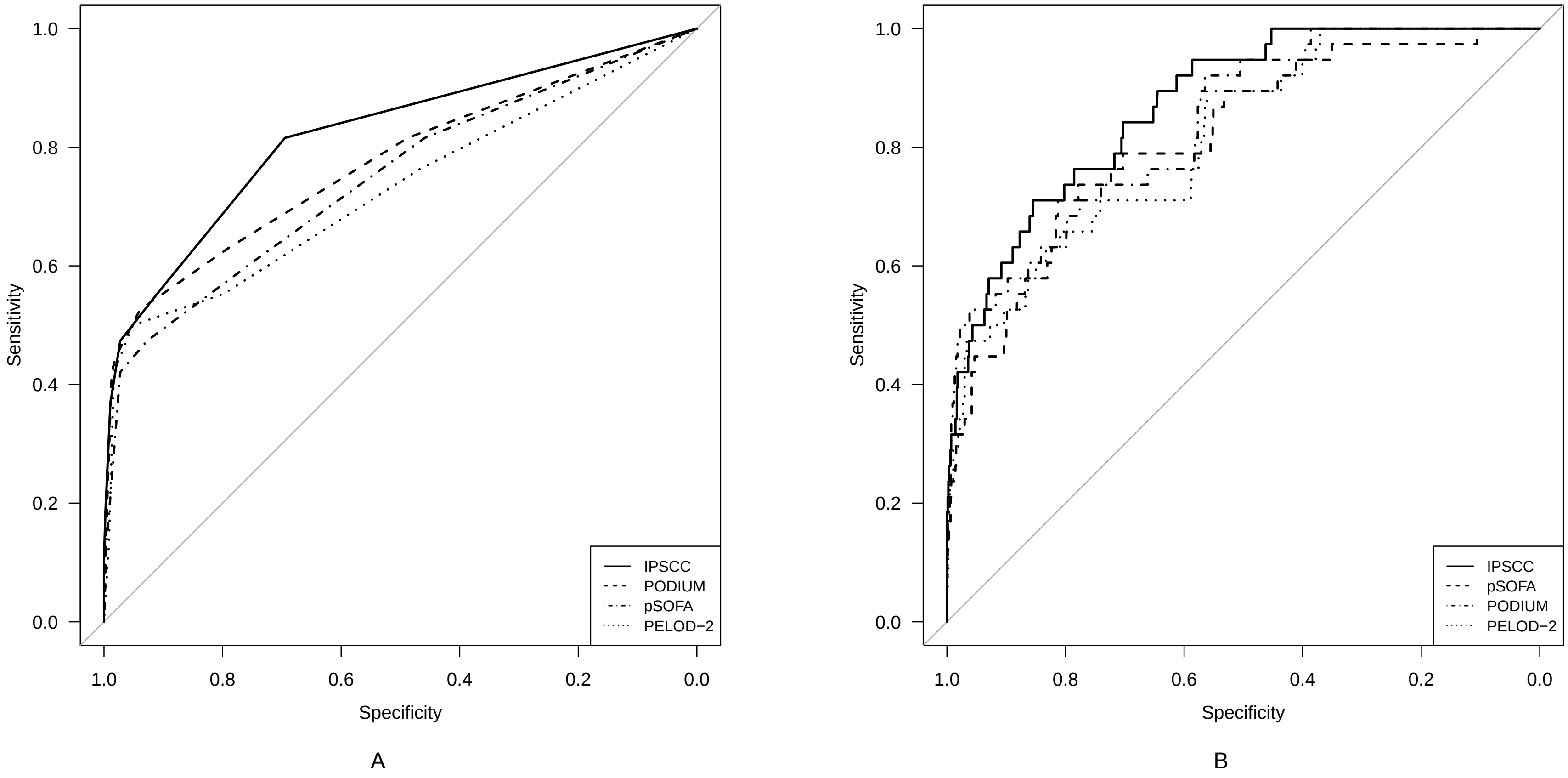

Figure S7: Receiver operating characteristics of the prediction of the secondary outcome based on organ dysfunction scores

Receiver operating characteristics curves based on unadjusted analyses (Panel A) and analyses adjusted for age (years), sex, and presence of a comorbidity (Panel B). IPSCC, International Pediatric Sepsis Consensus Conference; PELOD-2, Pediatric Logistic Organ Dysfunction-2; pSOFA, pediatric Sequential Organ Failure Assessment; PODIUM, Pediatric Organ Dysfunction Information Update Mandate.

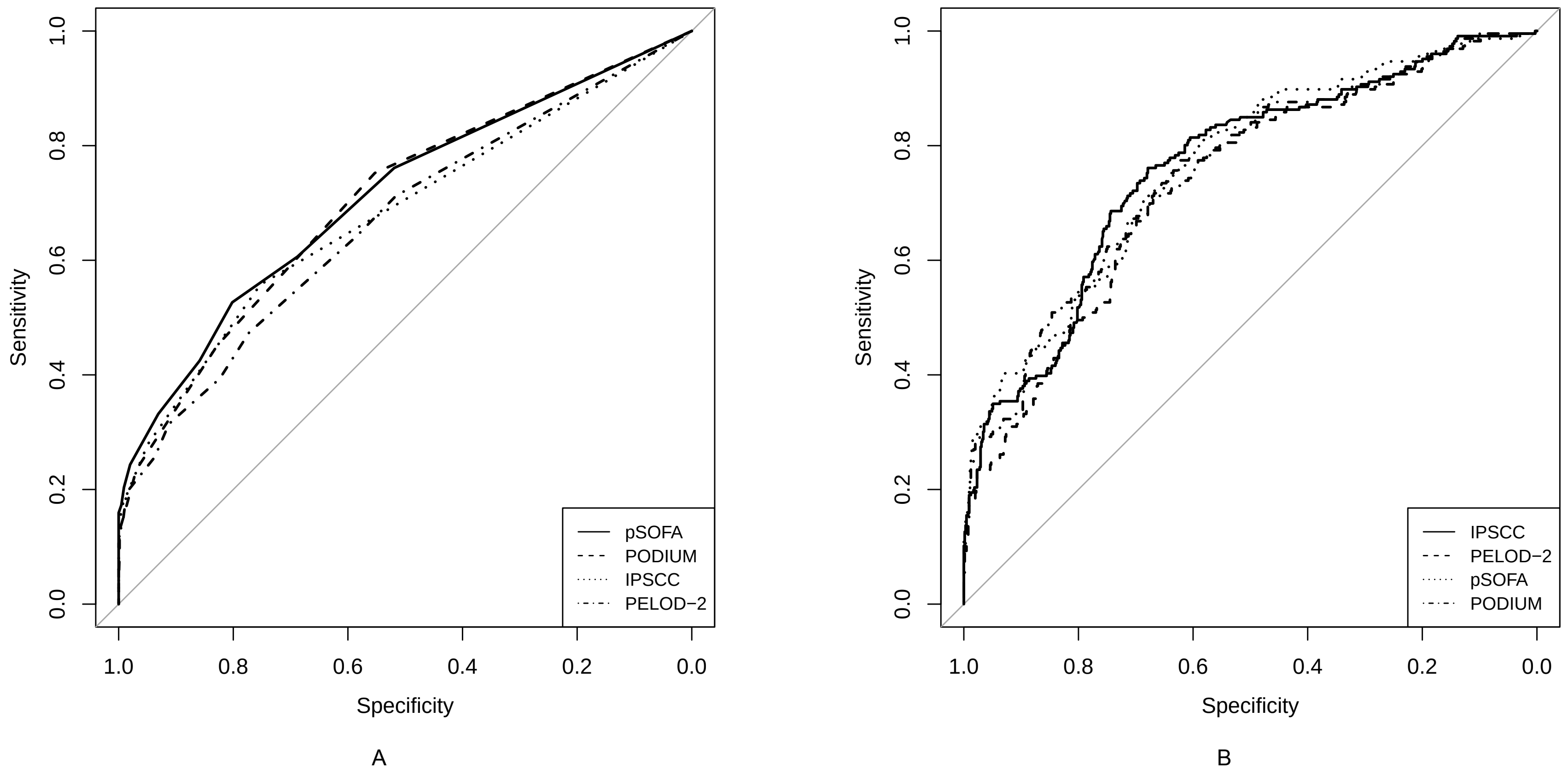

Figure S8: Receiver operating characteristics of the prediction of the secondary outcome based on "binarized" organ dysfunction scores

Receiver operating characteristics curves based on unadjusted analyses (Panel A) and analyses adjusted for age (years), sex, and presence of a comorbidity (Panel B). IPSCC, International Pediatric Sepsis Consensus Conference; PELOD-2, Pediatric Logistic Organ Dysfunction-2; pSOFA, pediatric Sequential Organ Failure Assessment; PODIUM, Pediatric Organ Dysfunction Information Update Mandate.

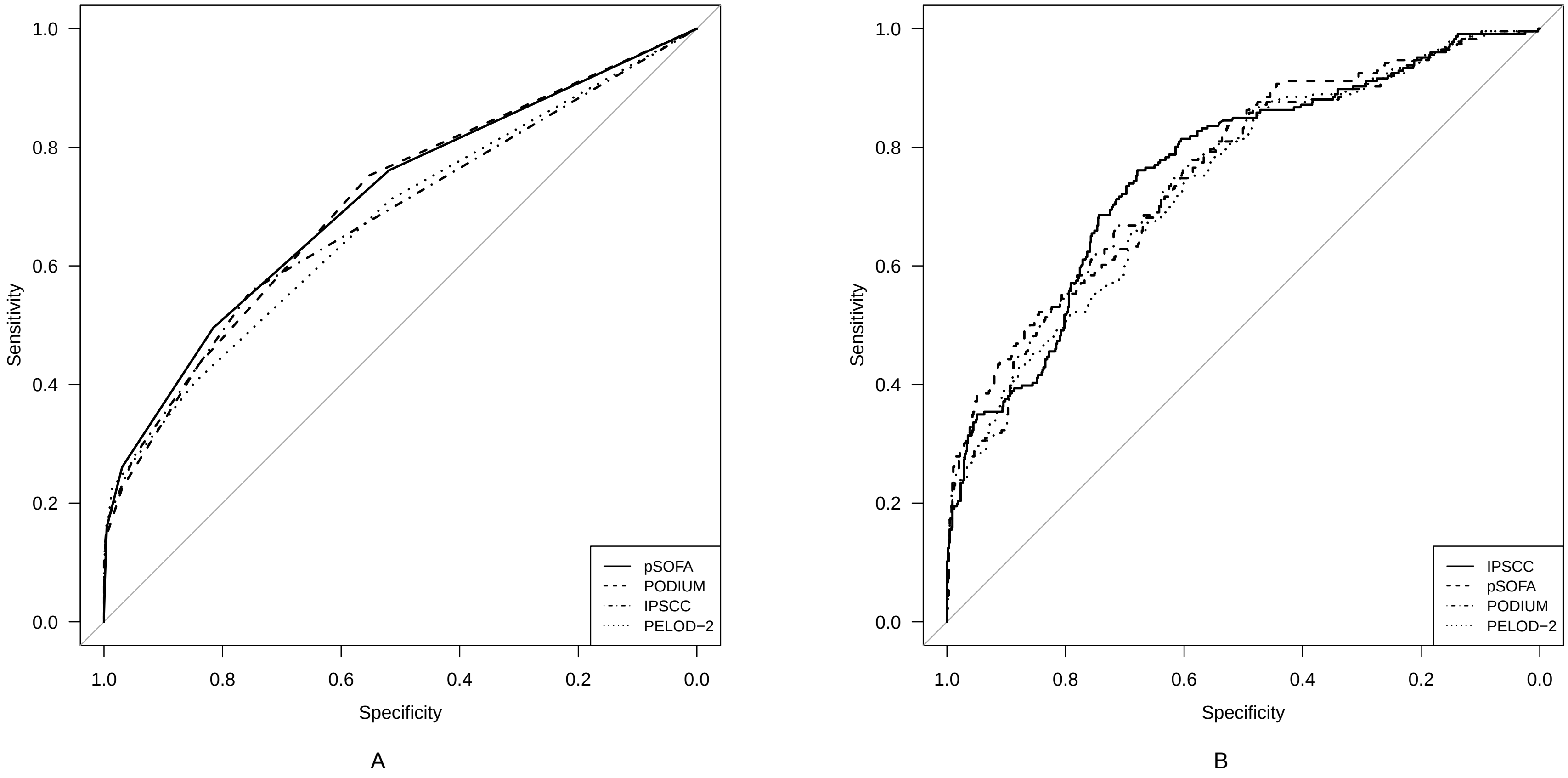

Figure S9: Importance of individual organ dysfunctions for the prediction of the primary outcome using only the first episode in each patient

Permutation importance of individual organ dysfunctions from conditional random forest analyses. Panel A, International Pediatric Sepsis Consensus Conference (IPSCC); Panel B, Pediatric Logistic Organ Dysfunction-2 (PELOD-2); Panel C, pediatric Sequential Organ Failure Assessment (pSOFA), and Panel D, Pediatric Organ Dysfunction Information Update Mandate (PODIUM). A larger value indicates higher importance compared to the other score items. Irrelevant covariates display permutation importance close to zero or negative values.

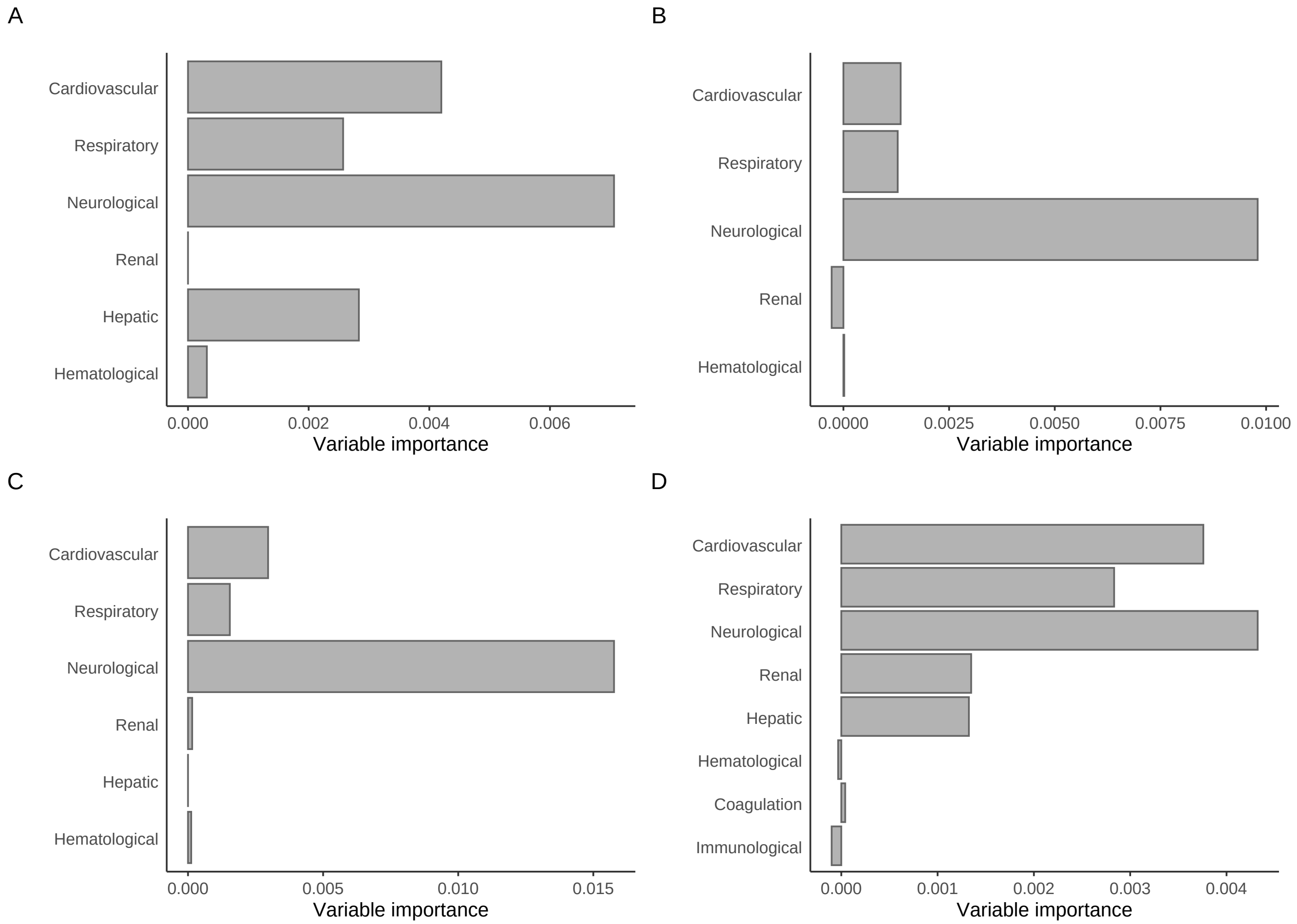

Figure S10: Receiver operating characteristics of the prediction of the primary outcome based on "simplified" organ dysfunction scores

IPSCC, International Pediatric Sepsis Consensus Conference; PELOD-2, Pediatric Logistic Organ Dysfunction-2; pSOFA, pediatric Sequential Organ Failure Assessment; PODIUM, Pediatric Organ Dysfunction Information Update Mandate

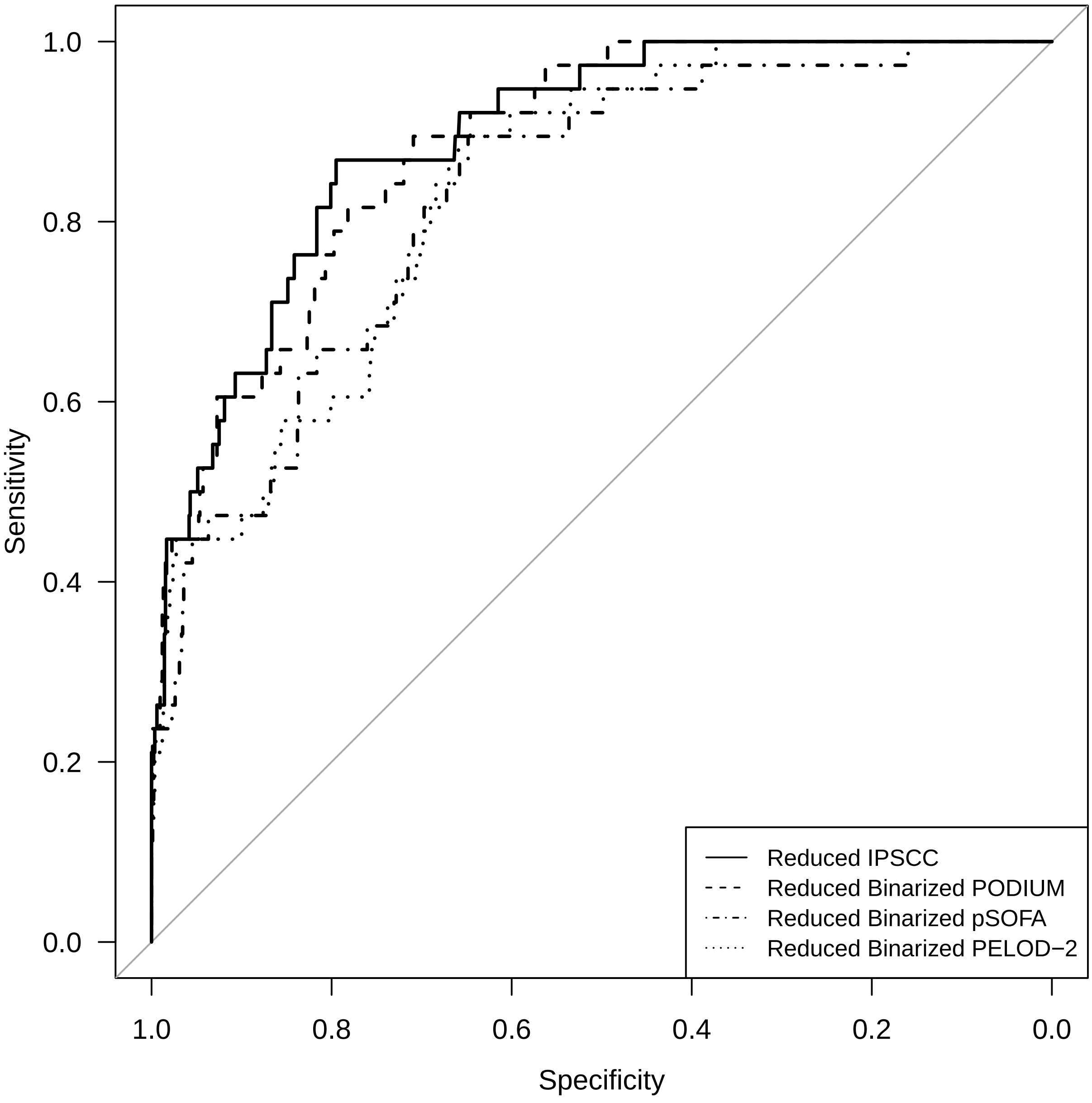

Figure S11: Receiver operating characteristics of the prediction of the secondary outcome based on "simplified" organ dysfunction scores

IPSCC, International Pediatric Sepsis Consensus Conference; PELOD-2, Pediatric Logistic Organ Dysfunction-2; pSOFA, pediatric Sequential Organ Failure Assessment; PODIUM, Pediatric Organ Dysfunction Information Update Mandate

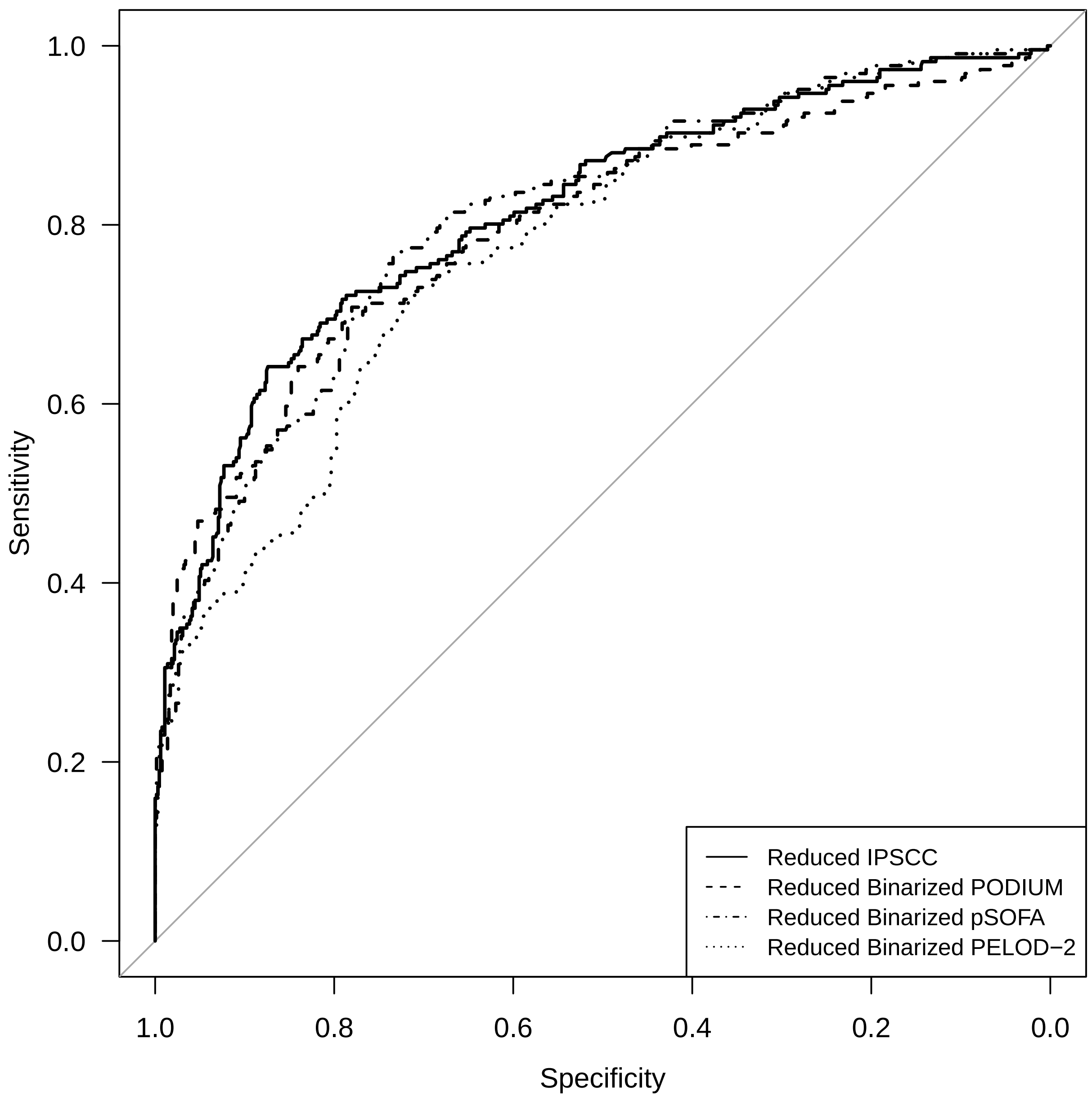

Supplement: Supplementary file 1 [file pcc-25-e117-s001.pdf]
